# Medicinal plants and natural products for treating overactive bladder

**DOI:** 10.1186/s13020-024-00884-3

**Published:** 2024-03-27

**Authors:** Huanxian Chen, Maggie Pui Man Hoi, Simon Ming Yuen Lee

**Affiliations:** 1 State Key Laboratory of Quality Research in Chinese Medicine, Institute of Chinese Medical Science, University of Macau, Macao, China; 2 Department of Pharmaceutical Sciences, Faculty of Health Sciences, University of Macau, Macao, China; 3 Department of Food Science and Nutrition, The Hong Kong Polytechnic University, Hong Kong, China

**Keywords:** Medicinal plants, Natural products, Overactive bladder

## Abstract

**Background:**

Overactive bladder (OAB) presents a high prevalence of 16–18% worldwide. The pathophysiology of OAB is still poorly understood while effective therapy or countermeasure are very limited. On the other hand, medicinal plants and herbal remedies have been utilized for treating lower urinary tract symptoms (LUTS) in both Eastern and Western cultures since ancient times. In recent years, accumulating progress has also been made in OAB treatment research by using medicinal plants.

**Methods:**

Relevant literature on the studies of medicinal plants and herbs used to treat OAB was reviewed. The medicinal plants were summarized and categorized into two groups, single-herb medications and herbal formulations.

**Results:**

The present review has summarized current understanding of OAB’s pathophysiology, its available treatments and new drug targets. Medicinal plants and natural products which have been used or have shown potential for OAB treatment were updated and comprehensively categorized. Studies on a wide variety of medicinal plants showed promising results, although only a few phytochemicals have been isolated and identified. Until now, none of these herbal compounds have been further developed into clinical therapeutics for OAB.

**Conclusions:**

This review provides the basis for discovering and designing new phytopharmaceutical candidates with effective and well-tolerated properties to treat OAB. Increasing evidences indicate new strategies with alternative herbal treatment for OAB have high efficacy and safety, showing great promise for their clinical use. Future studies in a rigorously designed controlled manner will be beneficial to further support the eligibility of herbal treatment as OAB therapeutics.

## Introduction

Lower urinary tract symptoms (LUTS) impact over 50% of the global adult population [82]. These symptoms emcompass storage, voiding, and post-micturition symptoms. Notably, overactive bladder (OAB) syndrome is classified as a specific subset within the domain of storage symptoms [2]. According to the International Continence Society (ICS), OAB describes the symptom complex of urinary urgency, often accompanied by increased frequency and nocturia, with or without urgency urinary incontinence, occurring in the absence of urinary tract infection or other obvious pathological coditions [54]. OAB is reconginized as a highly prevalent, troublesome and distressing condition. Its incidence tends to rise with age and it exerts a remarkable impact on quality of life (QOL). OAB affects both male and female equally, resulting in a large economic burden on individuals and society, in terms of the direct health care costs and lost productivity.

Presently, clinical strategies for pharmacotherapy of OAB are still limited to antimuscarinics and β3 agonists. Due to associated risks and adverse effects of conventional medicines, the use of alternative therapies to treat diseases nowadays has witnessed a rapid increase. Compared to synthetic chemicals, phytochemical compounds from medicinal plants are usually less expensive, less toxic and present less side effects. Ethnopharmacology has been applying traditional medicine and natural products for disease management. For instance, some currently used drugs, like aspirin, artemisinin, and digoxin, are deprived from plant extracts. Some regimens and single-herb medications of Traditional Chinese Medicine (TCM) have exhibited efficacy in managing symptoms associated with OAB. Natural phytochemicals enable the maintenance of a biological balance and avoid drug accumulation in the body [12]. Additionally, a remarkable increase in the use of medicinal plants and natural products to treat OAB has been observed. [12, 34, 96]. Herein, we review and summarize the body of evidence, obtained through traditional application and modern scientific methodology, which supports the use of medicinal plants, natural products and herbal formulations for the treatment of OAB. The summary and consolidation of the existing scientific data would greatly facilitate improvement of future research and offer OAB patients a wider range of potentially improved alternative therapies.

### Pathophysiology of OAB

**Fig. 1:**
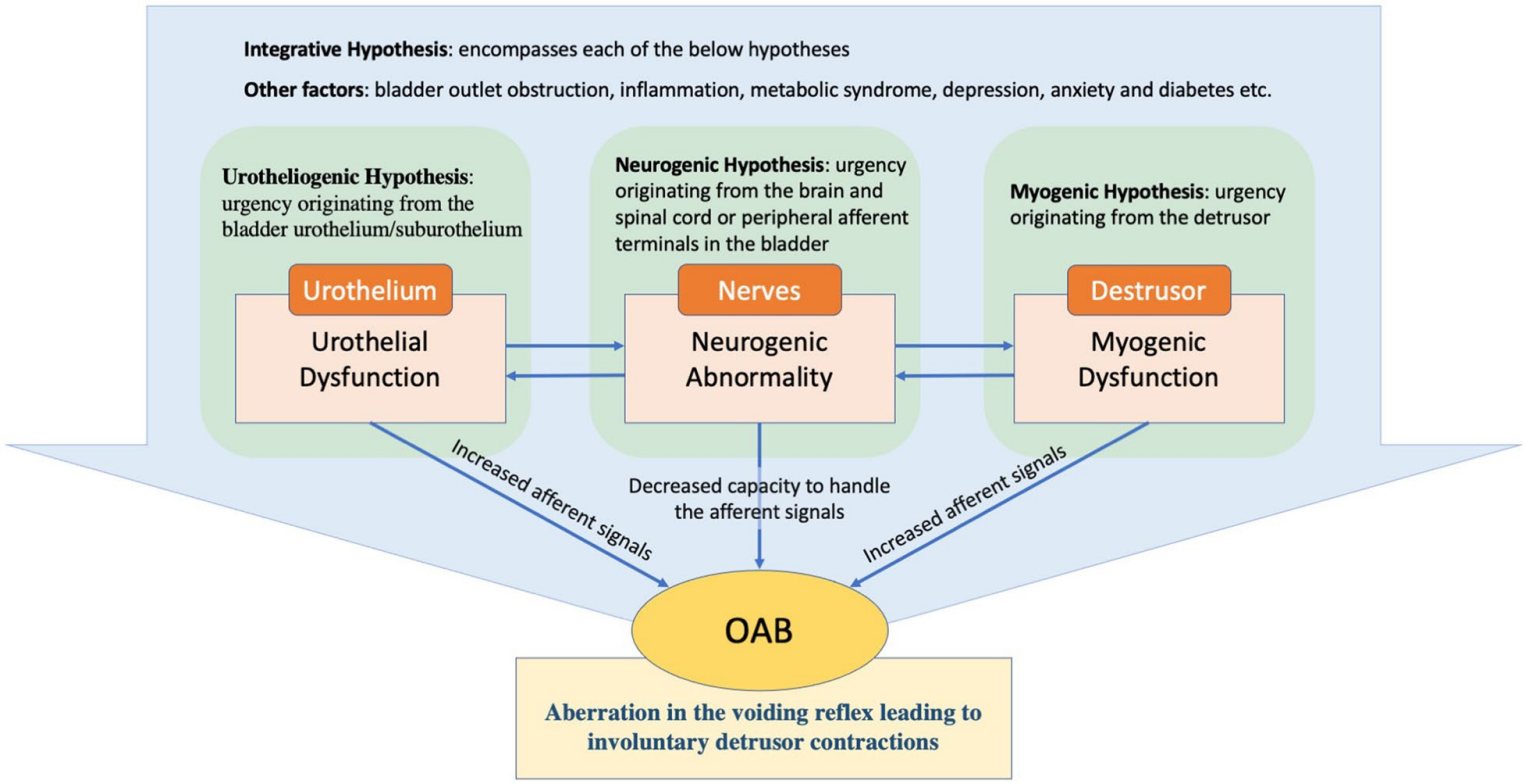
Pathophysiology and underlying mechanisms of OAB

At present, the etiology of OAB is largely considered idiopathic, and its underlying pathophysiology remains poorly comprehended, necessitating ongoing research endeavors. Two possible origins of OAB symptoms were proposed by the ICS: (1) reduced ability to process the afferent signals in the brain (the neurogenic hypothesis); and (2) abnormally enhanced afferent signals from the bladder and/or urethra: elevated afferent activity is considered to be associated either with aberration in the urothelium receptor function and neurotransmitter release (the urethrogenic hypothesis) or with aberration in myocyte excitability (the myogenic hypothesis) [123] (Fig. 1).

1. The urotheliogenic hypothesis

The urotheliogenic hypothesis refers to dysfunctions in signal molecules and ion channels within the urothelium. The urothelium not only acts as a protective barrier but also functions as a sensor to thermal, mechanical, and chemical stimuli. In the absence of a healthy urothelium, there may occur an elevation in spontaneous detrusor activity [107]. Through different receptors and transient receptor potential (TRP) channels, the urothelial cells respond to mechanical and chemical stimuli such as bradykinin, purines, norepinephrine, and acetylcholine (Ach), stimulating nearby afferent nerves. Abnormalities in the function of urothelium receptor and the release of neurotransmitter as well as in the sensitivity and coupling of the suburothelial interstitial result in involuntary contractions and manifestation of OAB symptoms [123, 166].

2. The myogenic hypothesis

The myogenic hypothesis posits that impaired function of the myocytes in the detrusor muscle may cause elevated excitability, hence leading to the occurrence of uncontrolled contractions [123]. Studies have demonstrated that myocytes obtained from the bladders of patients with detrusor overactivity (DO) display enhanced excitability and an amplified response to stimuli [23]. According to Drake et al. [37], it is hypothesized that DO could arise from histological alterations in the detrusor, resulting in abnormal electrical coupling of smooth muscle cells. Consequently, physiological micromotions get synchronised and transform into active involuntary contraction in the detrusor [59]. Bladder smooth muscle cells are interconnected through gap junction channels, predominantly composed of connexins, enabling their coupling. Although the involvement of connexins (Cxs) in the pathophysiology of OAB requires further study, Cx45 and Cx43 appear to be the most prominent Cxs expressed in human detrusor smooth muscle (DSM) cells [37, 111], and an increased Cx43 expression has been detected in individuals with neurogenic DO and urinary symptoms [8, 119].

3. The neurogenic hypothesis

The neurogenic hypothesis considers the abnormal management of afferent signals. In this case, urgency originates from the brain and spinal cord or peripheral afferent terminals in the bladder. Suprapontine lesions including cerebrovascular and neurodegenerative diseases like stroke and Parkinson’s disease, could cause aberrant central nervous system (CNS) activation and then lead to inappropriate excitation of the detrusor. Urinary retention occurs as a result of a spinal cord lesion, which eliminates voluntary and supraspinal control over the process of micturition [117, 123]. On the other hand, several specific neurotransmitters and related receptors participate in afferent signal transduction, such as muscarinic receptors and β3 adrenergic receptors. Among muscarinic receptors, M_2_ and M_3_ are the major subtypes expressed in the bladder [20]. Patients with idiopathic DO and painful bladder syndromes exhibit an elevated expression of M_2_ and M_3_ receptors in the bladder, and there is a notable correlation between the density of suburothelial M2 and M3 receptors and clinical urgency scores [109]. β3 adrenergic receptors is the dominant β-receptor subtype in the bladder [163], and expression of β3 adrenergic receptors in the bladder was strongly correlated with OAB-related symptoms [164].

4. The integrative hypothesis

The integrative hypothesis includes each of the above hypotheses since each of the components is likely to contribute to the pathophysiology of OAB considering the sophistication of mechanisms involved in micturition. Various potential triggers could induce local contractions (micromotions) in detrusor which are then transmitted to the bladder wall [35], resulting in occurrence of the sense of urgency. On the other hand, different other pathological and physiological conditions such as bladder outlet obstruction (BOO), inflammatory reactions, metabolic syndrome (such as diabetes), depression and anxiety also take part in the pathophysiology of OAB [106, 137]. Therefore, when patients present OAB symptoms, it is of utmost importance to conduct a comprehensive evaluation aimed at identifying and ruling out potential underlying causes.

### Current treatments for OAB

Various treatment options exist for OAB considering it involves multiple mechanisms. It is governed mainly by the severity of symptoms and the the extent to which it negatively affects patient’s quality of life (Table 1). General lifestyle changes have been suggested as a first-line therapy in all OAB patients; while pharmacotherapy or medications, including anticholinergics/antimuscarinics and β_3_ adrenergic agonists, are the mainstream treatment for OAB, based on their pharmacological efficacy both in theory and clinical application. They are regarded as second-line therapy for OAB. Third-line OAB therapy refers to neuromodulation of the nerves that control bladder function.

1. Lifestyle intervention, behavioral treatments and bladder training

**Table 1:** Therapy options for OAB [1, 94]

Level of therapy	Route	Treatment	Proposed mechanism of action
First-line therapy	Behavioral	Avoidance of bladder irritants, bladder retraining, pelvic floor physical therapy and biofeedback	Decreases irritation
Second-line therapy	Oral	Antimuscarinics	Suppresses detrusor muscle contractions
	Transdermal	Beta-3 Agonist	Relaxes detrusor smooth muscle
		Antimuscarinics	Suppresses detrusor muscle
Third-line therapy*	Neuromodulation	Sacral neuromodulation (SNS)	Unknown, electrically activates somatic afferent nerves in spinal nerve root
		Percutaneous tibial nerve stimulation (PTNS)	Unknown, electrically activates somatic afferent nerves in spinal nerve root
	Intravesical	Intradetrusor onabotulinumt oxin A (Botox^®^) injection	Paralyzes detrusor muscle

*May be offered to carefully selected patients who have failed first-line and second-line therapy, or who are not able to tolerate medications due to contraindications or adverse effects

The lifestyle intervention includes smoking termination, body weight reduction, timed voiding, modifying fluid intake, eliminating bladder irritants such as carbonated drinks and caffeine, adjusting bowel movements to avoid constipation and sprains during bowel movements and sleep [61, 101]. As for behavioral treatment, patients are trained to improve their ability to control and thereby disrupt or inhibit detrusor contraction. Pelvic floor muscle training (PFMT) could help inhibit pelvic floor contraction on the detrusor, therefore, ameliorating urgency and urge-related incontinence [99]. Bladder training is appled for cognitively and physically capable adults to further improve detrusor overactivity and regain continence by training them to gradually increase time interval between urinations [105, 121]. These practices mentioned must be accomplished daily with motivation and patience. If they fail to improve or control the symptoms, then pharmacotherapy or medications are added.

2. Anticholinergic or antimuscarinics drug

Drugs that inhibit acetylcholine-induced involuntary detrusor contractions are currently the mainstay treatment options for OAB. Anticholinergics were the first OAB pharmacotherapy on the market and present the largest available dataset [142]. It is widely recognised that normal bladder contraction in human is modulated primarily by stimulation or activation of muscarinic receptors within the detrusor muscle [17, 165]. The predominant cholinoceptors present in urinary bladder are M_2_ receptors, while its contraction is mainly mediated by the minor population of M_3_ receptors [165]. Therefore, M_3_-selective antimuscarinic agents (such as darifenacin and solifenacin) offer the first-line treatment for OAB, acting through competitively antagonizing acetylcholine at the M_3_ receptors in the DSM to inhibit DO [10]. However, regrettably, these M_3_-selective anti-muscarinic agents clinically result in various significant adverse reactiosns in many patients, including paralysis of accommodation, tachycardia, constipation, and dry mouth [11].

3. β3-adrenergic agonistic drug

During the urine storage stage in the bladder, norepinephrine (noradrenaline) is released by the sympathetic nerves and binds to the β3-adrenergic receptors on the DSM, exhibiting inhibitory action, which results in the bladder relaxation [43, 57]. Mirabegron is the only approved drug that acts as a effective and selective β3-adrenoceptor agonist. It is thought to relax the DSM by directly activating the β3 adrenergic receptors, which subsequently results in elevated cAMP and adenylyl cyclase levels in the tissue. In OAB rat models, mirabegron increases the average voiding volume in each urination, decreases the frequency of non-voiding contraction and enhances the bladder capacity without deranging discharge. Mirabegron could contribute to reduced annulled incidence; therefore, it can be applied in patients who discontinue previous anticholinergic/antimuscarinic therapy [14, 39, 124, 125, 141]. Common adverse effects of mirabegron include urinary tract infections, tachycardia, headache, and diarrhoea [150].

4. Neuromodulation and onabotulinum toxin A (Botox^®^) therapy

When patients fail first- and second-line OAB therapy, their conditions are regarded as refractory. Particularly, these patients show inadequate response to behavioral therapy. They also demonstrate either lack of response to medications (at least two types) or intolerance of medications (due to contraindications or adverse effects). Neuromodulation or onabotulinum toxin A (Botox^®^) therapy may be used for the carefully selected refractory patients. There are three different types of neuromodulation therapy, namely: peripheral tibial nerve stimulation (PTNS), sacral neuromodulation (SNS), and temporary chemodenervation of the bladder detrusor muscle. However, the treatment benefits are often counteracted by frequent and moderately severe adverse events such as lead migration, pain at the stimulator and lead sites, infection/irritation, electric shock, the requirement for additional surgeries, urinary tract infections (UTIs), dysuria, and hematuria etc. Onabotulinum toxin A (Botox^®^) therapy refers to injecting a chemodenervation agent, Botox^®^ (Allergan, Inc., Irvine, CA, USA), into the bladder, which was approved by FDA for idiopathic OAB in 2013. Onabotulinum toxin A is originated from a bacterium *Clostridium botulinum* and it binds to peripheral cholinergic terminals at the presynaptic membrane of the neuromuscular junction to inhibit acetylcholine release. This action causes muscle fibers paralysis until new fibers grow, thus temporarily affecting the myocytes in the bladder wall [94, 130, 153].

The current treatments for OAB present various limitations or side effects in clinical use. Hence, there is a strong rationale for the exploration and development of novel treatment strategies that aim to optimize therapeutic efficacy against OAB while simultaneously minimizing adverse reactions and side effects associated with effective dosages. Medicinal plants play an important role in this aspect since they have a long tradition in LUTS treatment. The screening and mechanistic elucidation of herbal drugs will greatly value OAB therapy.

### New drug targets for OAB treatment

Increasing research suggests that the etiology of OAB is multifactorial, necessitating the continued exploration of new drug candidates. Several new drug targets and cellular pathways in the urinary bladder to treat OAB have been proposed recently, as follow: (1) Modulators of cyclic nucleotide (cyclic adenosine monophosphate and cyclic guanosine monophosphate) that mediate adenosine triphosphate (ATP) release from bladder wall tissues, such as P2X3 receptor antagonists and nitric oxide (NO)-sensitive soluble guanylyl cyclase (sGC) activator; (2) new targets for β3 agonists, including the bladder muscularis mucosa and bladder wall blood vessels; (3) Various TRP channels (TRPV_1_, TRPV_4_, TRPM_8_, TRPA_1_, and TRPM_4_) and the effects of their mediators (antagonists) on detrusor overactivity; (4) Large and small conductance Ca^2+^‐activated K^+^ channels (BK and SK channels, respectively) and their impacts on spontaneous contractions; (5) Antioxidants that function to inhibit oxidative stress pathways; (6) Antifibrosis agents that directly or indirectly modulate the TGF‐β pathway, namely the canonical fibrosis pathway [9, 47, 158].

Recent research shows that medicinal plants and herbs could provide more efficacious treatment options with less side-effects. And they have been found to act on different targets or pathways compared to synthetic drugs. Current scientific evidence has demonstrated that a fair number of medicinal plants and natural products with efficacy of treating OAB act via one or a few of the targets mentioned above. Effects and mechanisms of various medicinal plants on OAB treatment from existing studies are summarized in Tables 2 and 3.

**Table 2:** Single-herb medications for overactive bladder treatment

Plant/herbal drug	Family	Common Name	Part used	Effect(s)	Study object(s)	Therapeutic target(s)/ mechanism(s)	References
*Alpinia oxyphylla*	Zingiberaceae	Sharp-leaf galangal, yì zhì 益智	Fruit (izalpinin)	Antagonized Carbachol-induced bladder strip contractions concentration-dependently	Rat bladder detrusor strips	Muscarinic receptor antagonistic action	[168]
*Artemisia monosperma*	Compositae	Wormwood	Aerial parts (7-O-methyleriodictyol)	Reduced bladder tone	Rat isolated bladder	NA	[3]
*Artemisia vulgaris*	Compositae	Common mugwort, wild wormwood, and felon herb	Aerial parts	Inhibited carbachol induced bladder contractions	Rabbit urinary bladder tissues/ strips	Mediation via dual, anticholinergic and Ca^2+^ antagonist mechanisms	[78]
*Aspalathus linaeris*	Fabaceae	Rooibos (meaning “red bush”)	Aerial parts	Inhibited carbachol induced bladder contractions	Rabbit urinary bladder tissues/ strips	Via dominant KATP channel opening and weak Ca^2+^ channel blockade pathways	[78]
*Astragalus membranaceus*	Leguminosae	Milkvetch or Huangqi, huáng qí 黄芪	Root (Astragalus polysaccharides, APS and Astragaloside I, AS-IV)	Ameliorated urinary frequency	CYP-induced OAB female mice model	Modulation of urothelial wound healing	[32]
*Bletilla striata*	Orchidaceae	Chinese ground orchid, bái jī 白芨	Pseudobulbs	Reduced AWR scores and amplitude of bladder detrusor‐EMG, extended micturition interval and increased storage of urine	Zymosan‐induced cystitis in female rats	NA	[97]
*Bridelia ferruginea*	Euphorbiaceae	Bridelia; Kirni, Kimi (Hausa), Maren (Fulani), Iralodan (Yoruba), and Oha, Ede, Ola in Igbo	Leaf (ethanol extracts)	Reduced KCl induced bladder contractile response	Rat bladder smooth muscle	Blockade of purinergic neurotransmission	[116]
*Bryophyllum pinnatum*	Crassulaceae	Airplant, life plant, Mother of Thousands, Miracle Leaf	Leaf	1. Reduced the electrically and carbachol-induced porcine DSM contractile response 2. Improved OAB symptoms, reduced micturition frequency in postmenopausal women 3. The flavonoid fraction decreased the porcine detrusor contractility dose- and time-dependently	1. Porcine detrusor strips 2. 20 postmenopausal female with OAB or urgency-dominant mixed urinary incontinence (MUI) 3. Porcine detrusor strips	1. NA 2. NA 3. NA	1. [129] 2. [18] 3. [48]
*Camellia sinensis*	Theaceae	Green tea	Leaf (EGCG)	1. Restored ovariectomy (OVX)-induced bladder dysfunction dose-dependently 2. Mitigated storage dysfunction, and protected the bladders from Metabolic syndrome (MetS) and OVX-induced interstitial fibrosis changes, alleviated bladder apoptosis	1. Surgical menopause-induced OAB rat model 2. Rat model with MetS and ovarian hormone deficiency	1. Antioxidant, anti-fibrosis and anti-apoptosis effects 2. Mitochondria and ER apoptosis pathways	1. [72] 2. [89]
*Cananga odorata*	Annonaceae	Ylang-ylang	Flower	Inhibited EFS and agonists induced urinary bladder contractile effects	In vitro: male Sprague–Dawley rats detrusor muscle strips In vivo: rabbit	c-AMP pathway	[79]
*Cannabis sativa*	Cannabaceae	Hemp	Whole plant (tetrahydrocannabinol, THC and cannabidiol, CBD)	1. Decreased urinary urgency, the number and volume of incontinence episodes, frequency and nocturia, improved patient self-assessment of pain, spasticity and quality of sleep 2. Reduced urge incontinence episodes 3. Reduced nicturia episodes, voids/ day, and the daytime voids, improved PGIC 4. Cannabidiol inhibited cholinergic-induced bladder smooth muscle contractions	1. 15 patients with advanced MS and refractory LUTS 2. 630 MS patients 3. 135 patients with MS and OAB 4. Strips cut from male Wistar rats and the human bladder body	1. NA 2. NA 3. NA 4. Modulation of TRPV1 in rats but not in humans	1. [24] 2. [46] 3. [76] 4. [26]
*Capsicum annuum*	Solanaceae	Chili pepper	Fruit	Improved clinical or urodynamic symptoms in 84.3% of the patients	A meta-analysis of 8 open and 2 placebo-controlled human clinical trials involving 200 patients with LUTS	NA	[36]
*Citrus depressa*	Rutaceae	Shekwasha	Fruit (Nobiletine)	Decreased micturition frequency	CYP-induced cystitis rats	The increase in intracellular cAMP in the bladder smooth muscles	[67]
*Cucurbita pepo*	Cucurbitaceae	Pumpkin	Seed (oil)	1. Reduced bladder pressure, increased bladder compliance, reduced urethral pressure 2. Reduced the degree of OABSS 3. Relieved BPH symptoms with no side effects	1. Rabbit 2. 45 volunteers (male: female = 25:20; age 41‐80 years) 3. 73 patients with BPH aged ≥ 50 years (single-blind randomized)	1. NA 2. NA 3. NA	1. [173] 2. [113] 3. [169]
*Cyclotrichium niveum*	Lamiaceae	Külotu (dag nanesi)	Aerial parts (essential oil)	Relaxed carbachol precontracted bladder strips	Rabbit bladder strip	NA	[29]
*Euphorbia resinifera*	Euphorbiaceae	African Spurge, Euphorbium, Resin Spurge	Resin (Resiniferatoxin, RTX)	1. Reduced bladder pain, and increased MCC; no significant improvement in frequency, nocturia, incontinence or FDC 2. Increased pain sensation, decreased bladder capacity, increased urinary frequency, and increased nociceptive behaviors	1. A meta-analysis of 7 trials of 355 patients with either IC or DO 2. Female Sprague–Dawley rats	1. NA 2. NA	1. [52] 2. [126]
*Galium aparine*	Rubiaceae	cleavers	Aerial parts	Traditionally used in cystitis	NA	NA	[25]
*Ganoderma lucidum*	Ganodermataceae	Reishi (in Japan) or líng zhī 灵芝 (in China) mushroom	1. Fruit body 2. Spore	1. Improved IPSS in men with LUTS 2. Counteracted the negative effects of ischemia/reperfusion (I/R) on bladder compliance and contractile responses	1. 50 male volunteers, ≥ 50 years old (double-blind, placebo-controlled randomized and dose-ranging) 2. In vivo I/R rabbit model	1. NA 2. Antioxidant effects	1. [114] 2. [90]
*Glycine max*	Fabaceae	Soybean	Seed (Daidzein and Genistein, phytoestrogens)	1. Genistein relaxed detrusor contracted with the muscarinic receptor agonist 2. A reduced risk of LUTS was associated with dietary intake of soy isoflavones 3. Reduced detrusor contractions induced by EFS dose-dependently	1. Isolated strips of rabbit detrusor 2. A Cross-sectional study on 2000 elderly Chinese men 3. Whole rat urinary bladders: in vivo treatment, ex vivo contractility examination	1. Inhibition of voltage operated Ca^2+^ channels (VOCCs) 2. NA 3. Activation of large and small conductance K^+^(Ca) channels	1. [120] 2. [155] 3. [147]
*Hippophae rhamnoides*	Elaeagnaceae	Seaberry, sea buckthorn	Fruit	1. Ursolic acid and isorhamnetin 7-O-rhamunoside inhibited Carbachol and TGF-β-induced constriction 2. Improved emotional parameters associated with urinary dysfunction	1. Rat bladder muscle strips; human bladder smooth muscle cells 2. Japanese male and female with mild urinary dysfunction	1. Ursolic acid and related compounds in seaberry extract may directly bind to the TGF-β receptor 2. NA	1. [132] 2. [140]
*Hypericum perforatum*	Hypericaceae	St John’s wort	Flowering top	Inhibited EFS-induced contractility	Rat urinary bladder strips	The inhibitory effect on excitatory transmission involve opioid receptors, at least partly	[27]
*Perilla frutescens*	Lamiaceae	Beefsteak plant	Leaf	Increased in the micturition interval, improved frequent urination	Female SHR rats	Improvement of the urothelial presence, anti-inflammatory effects	[84]
*Peucedanum japonicum*	Apiaceae	Coastal hog fennel	Whole plant extract / Isosamidin	1. The extract inhibited agonist-induced rabbit bladder contractile response; decreased micturition frequency 2. Isosamidin inhibited phenylephrine‐stimulated contractions dose‐dependently 3. The extract improved IPSS-QOL score; improved nocturia, and OABSS-2	1. Rabbit bladder strips; acetic acid-induced hypertensive bladder in rats 2. Human bladder and prostate strips from 9 to 10 male patients 3. 20 patients (male, age ≥ 50 years, with untreated LUTS, and no serious complications	1. NA 2. NA 3. NA	1. (Ito Y, 2013) 2. [139] 3. [74]
*Potentilla chinensis*	Rosaceae	Chinese Cinquefoil	Aerial part (aqueous extract)	1. Attenuated RA-induced DO in rats 2. Attenuated DO in rats with hemorrhagic cystitis	1. RA-induced DO in rats 2. CYP-induced hemorrhagic cystitis in rats	1. Inhibition on the release of transmitters from afferent and efferent fibres; influence on the exocytotic process depending on SNARE protein activity 2. Inhibition on the release of transmitters from afferent and efferent fibres; antioxidant effects	1. [156] 2. [73]
*Puerariae lobatae*	Leguminosae	Kudzu, Gegen, gé gēn 葛根	Dried root (water extract)	1. Induced detrusor relaxation in urothelium- independent manner 2. Improved DSM overactivity	1. Isolated rat bladder strips 2. Male SHR rats	1. NA 2. Neurogenic and anti-muscarinic (M3 receptor) mechanisms	1. [92] 2. [175]
*Rhois aromatica*	Anacardiaceae	Sweet sumach bark	Whole plant	Inhibited Carbachol and KCl-induced contractions	Rat and human bladder	Direct antagonistic effect on muscarinic receptors	[21]
*Salvia cinnabarina*	Lamiaceae	Cinnabar Sage	Aerial parts (3,4-secoisopimar-4(18),7,15-triene-3-oic acid)	Inhibited EFS-contratile response in a dose-dependent manner	Male Wistar rat bladders	NO production	[28]
*Serenoa repens*	Palmae	Saw palmetto	Fruit	1. Improved bladder voiding and LUTS, IPSS and NIH-CPSI, as well as erectile function 2. Decreased contractility and hyperplasia, improved smooth muscle fiber structure and reduced cell proliferation in the bladder 3. Improved IPSS-QOL score, nocturia, and OABSS-2 4. Alleviated daytime frequency and nocturia 5. NA	1. 591 patients with chronic benign prostate conditions related to inflammation 2. Obese male Wistar rats induced by high-carbohydrate diet 3. 20 male patients with untreated LUTS, and no severe complications, age ≥ 50 years 4. 76 adult women with urinary symptoms 5. NA	1. NA 2. NA 3. NA 4. NA 5. Antiandrogenic action, anti-inflammatory/anti-oedematous effect, prolactin signal modulation in benign prostatic hyperplasia	1. [51] 2. [41] 3. [74] 4. [162] 5. [50]
*Silybum marianum*	Asteraceae	Silymarin	Seed	Reduced contractile response in cystitis	CYP-induced cystitis rat model	Antioxidant and anti-inflammatory effects	[40]
*Solanum lycopersicum*	Solanaceae	Saladette tomato	Fruit (lipidic extract)	Decreased contractility and hyperplasia, improved smooth muscle fiber structure and reduced cell proliferation in the bladder	High-carbohydrate diet induced obese Wistar rats (male)	NA	[41]
*Solidaginis virgaurea*	Asteraceae	European golden rod	Whole plant	Inhibit Carbachol and KCl-induced contractions	Rat and human bladder strips	Direct antagonistic effect on muscarinic receptors	[21]
*Uncariae Ramulus Cum Uncis*	Rubiaceae	Gambir Plant, gōu ténɡ 钩藤	Branch with hooks (Rhynchophylline)	1. Inhibited the contraction of urinary bladder strips 2. Reduced the maximum bladder capacity, bladder filling pressure, leak point pressure, contraction frequency, and motility index	1. Isolated rat urinary bladder strips 2. Rats with infravesical outflow obstruction	1. Blockade of L-type calcium channel and activation of calcium-activated potassium channel 2. Blockade of L-type calcium channels and activation of big- conductance calcium-activated potassium channels	1. [70] 2. [71]
*Vaccinium corymbosum*	Ericaceae	Blueberry	Fruit	Prevented the progression of bladder dysfunction resulting from BOO	BOO rats	Antioxidation and the inhibition of bladder remodeling	[108]
*Vanilla planifolia*	Orchidaceae	Vanilla	Fruit (oil)	Vanilla oil reduced serum catecholamine levels and urination frequency in rats under light urethane anesthesia	Sprague–Dawley rats	Reduction of sympathetic activity	[138]
*Vitis vinifera*	Vitaceae	Grape	Fruit (grape suspension, Resveratrol)	1. Protective effects on the decreased contractile response of isolated bladder strips to a field stimulation (FS) induced by H_2_O_2_ 2. Reversed increases in non-voiding contractions, post-voiding pressure and voiding frequency; lowered ROS levels and serum lipid per-oxidation in bladder tissues 3. Resveratrol improves OAB	1. Male rabbit bladders 2. Obese mice 3. Rats with chronic prostatitis	1. Protection of the citrate synthase to the oxidative effects of H_2_O_2_ activity of the muscle 2. Anti-oxidant effects 3. Downregulation of the protein expression of SCF, c-Kit and p-AKT	1. [44] 2. [6] 3. [167]
*Zea mays*	Poaceae	Purple corn	Flowering top (stigma), corn silk	Traditionally used in cystitis	NA	NA	[30]

*NA* not available

**Table 3:** Herbal formulations for overactive bladder treatment

Formulation	Plant/herbal drug	Family	Common name	Part used	Effect	Study object(s)	Therapeutic target(s)/ mechanisms	References
**TCM formulations (Proprietary Chinese medicine)**								
Bu-Zhong-Yi-Qi-Tang (补中益气汤)	*Astragalus membranaceus*	Leguminosae	Milkvetch or huáng qí 黄芪	Roots	1. Co-treatment with BZYQT and SPXS significantly improved the frequency of voiding, urgency and urge incontinence as well as increased the voiding volume and QOL 2. Significantly improved OABSS and QOL after 4 weeks treatment 3. Co-treatment with BZYQT and propiverine improved OAB symptoms and showed better efficacy than treatment with propiverine alone	1. 21 OAB patients 2. 28 female OAB patients with pattern of collapse from Qi deficiency 3. 34 patients with OAB with Qi deficency syndrome	NA	1. [95] 2. [91] 3. [33]
	*Atractylodes macrocephala*	Asteraceae	bái zhú 白术	Roots				
	*Panax ginseng*	Araliaceae	Ginseng, rén shēn 人参	Root				
	*Angelica sinensis*	Apiaceae	Chinese Angelica Root, dāng guī 当归	Root				
	*Bupleurum chinense*	Apiaceae	Bupleurum, hare’s ear root, thorowax root, chái hú 柴胡	Root				
	*Ziziphus zizyphus*	Rhamnaceae	Jujube fruit, Chinese date, dà zǎo 大枣	Fruit				
	*Citrus reticulata*	Rutaceae	mandarin orange, chén pí 陈皮	Aged mandarin orange peel				
	*Glycyrrhiza uralensis*	Leguminosae	Chinese liquorice, gān cǎo 甘草	Roots				
	*Cimicifuga foetida*	Ranunculaceae	Foetid Bugbane, shēng má 升麻	Rhizome				
	*Zingiber officinale*	Zingiberaceae	Ginger, shēng jiāng 生姜	Rhizome				
Ba-Wei-Di-Huang-Wan (八味地黄丸); Hachi-mi-jio-gan	*Rehmannia glutinosa*	Scrophulariaceae	Chinese foxglove, dì huáng 地黄	Root tuber	1. Lowered plasma glucose level, partially reversed the increases in contractile response to APE and the M2 receptor protein density 2. Inhibited acetylcholine-induced contraction 3. Ameliorated the reduced voiding interval, micturition volume, and bladder capacity induced by ATP solution 4. Ameliorated CYP-induced ongoing bladder overactivity and acidic ATP solution-induced hyperactivity on rats’ prestimulated bladder 5. Depressed SP-enhanced pelvic afferent nerve activity, bladder NF-kB/ICAM-1 expression, leukocyte infiltration, and ROS amount, and alliveated bladder hyperactivity	1. Streptozotocin-induced diabetic rats 2. Isolated rat bladder strips 3. Female SHRs 4. Female Wistar rats with CYP-induced ongoing bladder overactivity 5. Exogenous SP-induced bladder hyperactivity in female Wistar rats	1. Alleviation of M2 receptor overexpression 2. Binding to muscarinic receptors, 1,4-DHP receptors and purinergic receptors 3. Reduction in expression of tachykinins and P2X3 and TRPV1 receptors 4. Inhibition of the overexpression of mucosal P2X2, P2X3, M2 and M3-mAChR protein, as well as detrusor M2 and M3-mAChR protein; prevention of TRPV1 and P2X3 receptor overexpression 5. Inhibition of SP/neurokinin-1 receptor and NF-kB/ICAM-1 signaling pathway	1. [144] 2. [66] 3. [63] 4. [88] 5. [145]
	*Cornus officinalis*	Cornaceae	Asiatic dogwood, Japanese cornel, shān zhū yú 山茱萸	Dry ripe sarcocarp				
	*Dioscorea opposita*	Dioscoreaceae	Chinese yam, shān yào 山药	Tuber or root				
	*Alisma orientale*	Alismataceae	Asian water plantain, zé xiè 泽泻	Rhizome				
	*Poria cocos (Schw.) Wolf*	Polyporaceae	Hoelen, China root, fú líng 茯苓	Filaments under the cap				
	*Paeonia suffruticosa*	Paeoniaceae	Moutan peony, mǔ dān pí 牡丹皮	Root bark				
	*Cinnamomum verum*	Lauraceae	Cinnamon, guì pí 桂皮	Dried bark				
	*Aconitum carmichaelii*	Ranunculaceae	Chinese aconite, Carmichael’s monkshood, fù zǐ 附子	Dried root				
Fangjihuangqi Tang (防己黄芪汤)	*Stephania tetrandra*	Menispermaceae	Stephania-root, fáng jǐ 防己	Roots	Relieved symptoms of lower urinary tract dysfunction	BPH rat model	Regulation of smooth muscles in the bladder and urethra	[31]
	*Astragalus membranaceus*	Leguminosae	Milkvetch or huáng qí 黄芪	Roots				
	*Atractylodes macrocephala*	Asteraceae	bái zhú 白术	Roots				
	*Glycyrrhiza uralensis*	Leguminosae	Chinese liquorice, gān cǎo 甘草	Roots				
Ji‐Sheng‐Shen‐Qi‐Wan (济生肾气丸); Gosha-jinki-gan	Ba-Wei-Di-Huang-Wan				1. Enhanced threshold pressure, voiding interval, micturition volume, and bladder capacity 2. Increased intercontractile intervals and decreased contraction amplitudes; decreased dopamine and serotonin levels in plasma 3. Prevented overexpression of tachykinins and TRPV1 and P2X3 receptors in bladder urothelium 4. Reduced both episodes of nocturia and the IPSS	1. AA induced bladder overactivity in female SD rats 2. AA induced OAB in female rats 3. AA induced detrusor overactivity in female rats 4. 30 patients with nocturia despite treatment with α1-blockers or antimuscarinic drugs for at least 4 weeks	1. Inhibition of resiniferatoxin (RTX)-sensitive afferent neurons 2. Possible effects on the afferent and efferent micturition reflex and central effects on micturition mechanisms 3. Reduced expression of transmitter proteins and sensory receptors without damaging nerve fibers 4. NA	1. [172] 2. [112] 3. [62] 4. [161]
	*Achyranthes japonica*	Amaranthaceae	Oriental chaff flower or Japanese chaff flower, niú xī 牛膝	Root				
	*Plantago asiatica*	Plantaginaceae	Chinese plantain, Asiatic plantain, chē qián zǐ 车前子	Dried ripe seed				
Modified Ojayeonjonghwan (Wuzi Yanzong wan)	*Cornus officinalis*	Cornaceae	Asiatic dogwood, Japanese cornel, shān zhū yú 山茱萸	Dried sarcocarp	Similar pharmacologic effects to solifenacin in controlling DO caused by BOO	Partial urethral obstruction-induced DO rat model	Anti-inflammatory effect and antioxidant effects, enhancement of the NO pathway	[15]
	*Lycium chinense*	Solanaceae	Goji Berry, Chinese Wolfberry, gǒu qǐ 枸杞	Fruit				
	*Rubus coreanus*	Rosaceae	Korean blackberry, or Korean bramble, fù pén zǐ 覆盆子	Fruit				
	*Cuscuta chinensis*	Convolvulaceae	Chinese Dodder, tù sī zǐ 菟丝子	Dry seed				
	*Schisandra chinensis*	Schisandraceae	magnolia-vine, magnolia berry or five-flavor-fruit, wǔ wèi zi 五味子	Fruit				
Sang Piao Xiao San (Mantis Formula, 桑螵蛸散)	*Mantidis Ootheca*	Mantidae	Mantis Egg Capsule, Mantis Egg Case	Not a plant, egg chamber	1. Co-treatment with BZYQT and SPXS significantly reduced the frequency of voiding, urgency and urge incontinence as well as improved the voiding volume and QOL 2. Co-treatment with solifenacin improved TCM symptom scores, the scores of urination, nocturnal urination, urgency of urination and urgency incontinence, compared to treatment with solifenacin alone. It improved bladder compliance, maximum urinary flow rate and maximum bladder capacity, the initial urine volume (VFD), and also lowered NGF and NGF/Cr level to a greater extent than using solifenacin alone	1. 21 OAB patients 2. 128 patients with OAB after menopause	NA	1. [95] 2. [98]
	*Polygala tenuifolia Willd*	Polygalaceae	yuǎn zhì 远志	Root				
	*Acorus gramineus*	Acoraceae	Grassy-leaved sweet flag, shí chāng pú 石菖蒲	Root				
	*Fossilia Ossis Mastodi*	NA	Dragon’s Bone, lóng gǔ 龙骨	Not a plant, The fossilized bone or vertebrae				
	*Panax ginseng*	Araliaceae	Ginseng, rén shēn 人參	Root				
	*Poria cocos (Schw.) Wolf*	Polyporaceae	Poria Mushroom with Hostwood, fú shén 茯神	Sclerotium				
	*Angelica sinensis*	Apiaceae	Chinese Angelica Root, dāng guī 当归	Root				
	*Carapax Testudinis et Plastrum*	Emydidae	Tortoise Carapace and Plastron	Not a plant, carapace and plastron of Chinemys reevesii (Gray)				
Suo-Quan-Wan (缩泉丸)	*Alpinia oxyphylla Miq*	Zingiberaceae	Sharp-leaf galangal, yì zhì rén 益智仁	Fruit	1. Slowed down OAB progress and improved overall bladder function, recovered bladder stability 2. Improved bladder control, storage and contraction ability 3. Enhanced urodynamic urination, attenuated thickened bladder wall, decreased DSM strips contraction response for stimuli	1. BOO rats model 2. Aging rats 3. Diabetic mice	1. Decrease of TRPV1 expression 2. Increase in the sensitivity and expression of β3-AR 3. Mediation of the expression levels of myosin Va and SLC17A9	1. [85] 2. [160] 3. [151]
	*Dioscorea opposita*	Dioscoreaceae	Chinese yam, shān yào 山药	Tuber or root				
	*Lindera strychnifolia*	Lauraceae	Uyaku, wū yào 乌药	Root				
Wenglitong capsule (翁沥通胶囊)	*Coix lachryma-jobi*	Poaceae	Job’s tears, adlay or adlay millet, yì yǐ rén 薏苡仁	Seed	Improved OABSS, voiding frequency, average voided volume and urgency incontinence; slower onset and weaker efficacy but lower incidence of side effects. than tolterodine. Cotreatment of WLT and tolterodine was more effective than tolterodine alone in ameliorating OAB symptoms	182 female OAB patients (A prospective, randomized, single-blind multi-center trial)	NA	[159]
	*Fritillaria thunbergi Bulbus*	Liliaceae	Chekiang Fritillary Bulb, zhè bèi mǔ 浙贝母	Bulb				
	*Clematis armandii*	Ranunculaceae	Armand Clematis Stem, chuān mù tōng 川木通	Dried lianoid stem				
	*Gardenia jasminoides*	Rubiaceae	Cape jasmine, zhī zǐ 栀子	Flower				
	*Lonicera japonica*	Caprifoliaceae	Golden-and-silver honeysuckle, jīn yín huā 金银花	Flower bud				
	*Inula japonica*	Asteraceae	Japanese Inula Flower, xuán fù huā 旋覆花	Flower				
	*Lycopi Herba*	Lamiaceae	Hirsute Shiny Bugleweed Herb, zé lán 泽兰	Whole plant				
	*Mineralium Viridianum*	(not a plant)	Verdigris, tóng lǜ 铜绿	NA				
	*Glycyrrhiza uralensis*	Leguminosae	Chinese liquorice, gān cǎo 甘草	Roots				
	*Astragalus membranaceus*	Leguminosae	Milkvetch or huáng qí 黄芪	Roots				
	*Rheum officinale*	Polygonaceae	Chinese rhubarb or dà huáng 大黄	Roots				
**Non-TCM formulations**								
Choreito (CRT)	*Aluminum sili-cate hydrate with silicon dioxide*	(Not a plant)	NA	NA	Attenuated DO	DO induced by intravesical AA instillation in rats	Remission of urothelial damage and regulation of excess blood flow	[146]
	*Alisma orientale*	Alismataceae	Asian water plantain, zé xiè 泽泻	Rhizome				
	*Polyporus umbellatus*	Polyporaceae	lumpy bracket, umbrella polypore	Sclerotium				
	*Poria cocos*	Polyporaceae	Hoelen, China root, fú líng 茯苓	Sclerotium				
	*Donkey glue*	( Not a plant)	NA	NA				
Eviprostat	*Equisetum arvense*	Equisetaceae	Field horsetail or common horsetail	Ethanol extract	1. Relieved obstructive symptoms of BPH; improved prostatic volume, urinary flow rates and prostatic inflammation 2. Improved IPSS, QOL score, Qmax and Qave 3. Decreased the urinary oxidative stress marker 8-OHDG in a PBOO rabbit model; lowered urinary 8-OHdG levels, reduced IPSS and QOL score in a clinical trial 4. Suppressed the increase of oxidative-stress markers, and pro-inflammatory cytokine levels in bladder; reversed the decrease in the intermicturition interval 5. Prevented the reduction in BBF and increases in bladder weight, malondialdehyde levels, proinflammatory cytokines, and myeloperoxidase activity 6. Improved DO, down-regulated the expression of bladder pharmacological receptors and up-regulated urinary cytokine levels	1. 22 patients with symptomatic BPH 2. 100 patients with BPH 3. A rabbit model of Partial BOO and 9 BPH/LUTS patients 4. A rat model of atherosclerosis-induced chronic bladder ischemia 5. A rat model of bladder overdistension and emptying (OE) 6. Rats with CYP induced cystitis	1. NA 2. NA 3. Antioxidant effect 4. Antioxidant and anti-inflammatory properties 5. Antioxidant and anti-inflammatory effects 6. Anti-inflammatory effects	1. [64] 2. [135] 3. [104] 4. [103] 5. [77] 6. [110]
	*Pulsatilla pratensis*	Ranunculaceae	Pasque Flower	Ethanol extract				
	*Populus tremula*	Salicaceae	Aspen, quaking aspen	Ethanol extract				
	*Chimaphila umbellata*	Ericaceae	Umbellate wintergreen	Ethanol extract				
	*Triticum aestivum*	Poaceae	Common wheat	Germ oil				
Granu Fink femina	*Cucurbita pepo*	Cucurbitaceae	Uromedic pumpkin	Seed oil	Improved all measured aspects of OAB-related QOL after 1 week, with further improvement at 6 and 12 week	117 female (age: 21–78) with OAB	NA	[49]
	*Rhus aromatica*	Anacardiaceae	Fragrant sumac	Bark extract				
	*Humulus lupulus*	Cannabaceae	Common hop	Cone extract				
Kubiker	*Cucurbita maxima*	Cucurbitaceae	Winter squash	Fruit	Lowered daily micturitions, nocturia and episodes of urge incontinence, improved PPIUS, OAB-q SF and PGI-I, in a greater level than Solifenacin Succinate	90 consecutive female (mean age 65 years; range 40–75) with symptoms of OAB	NA	[148]
	*Capsicum annuum*	Solanaceae	Chili Pepper	Fruit				
	*Polygonum capsicatum*	Polygonaceae	Knotweed, knotgrass	Root and rhizome				
	*Vitamins (C and D)*	(Not a plant)	NA	NA				
	Amino acid l-Glutammina	(Not a plant)	NA	NA				
Urox	*Crataeva nurvala*	Capparaceae	Three leaved caper	Stem bark	Reduced urinary day frequency, episodes of nocturia, symptoms of urgency, total incontinence; improved quality of life, with minimal side-effects	150 participants (Phase-2, parallel double-blinded, randomized clinical study)	NA	[128]
	*Equisetum arvense*	Equisetaceae	Horsetail	Stem				
	*Lindera aggregata*	Lauraceae	Spicebush, wū yào 乌药	Root				

*NA* not available

### Medicinal plants and natural products for OAB treatment

**Fig. 2:**
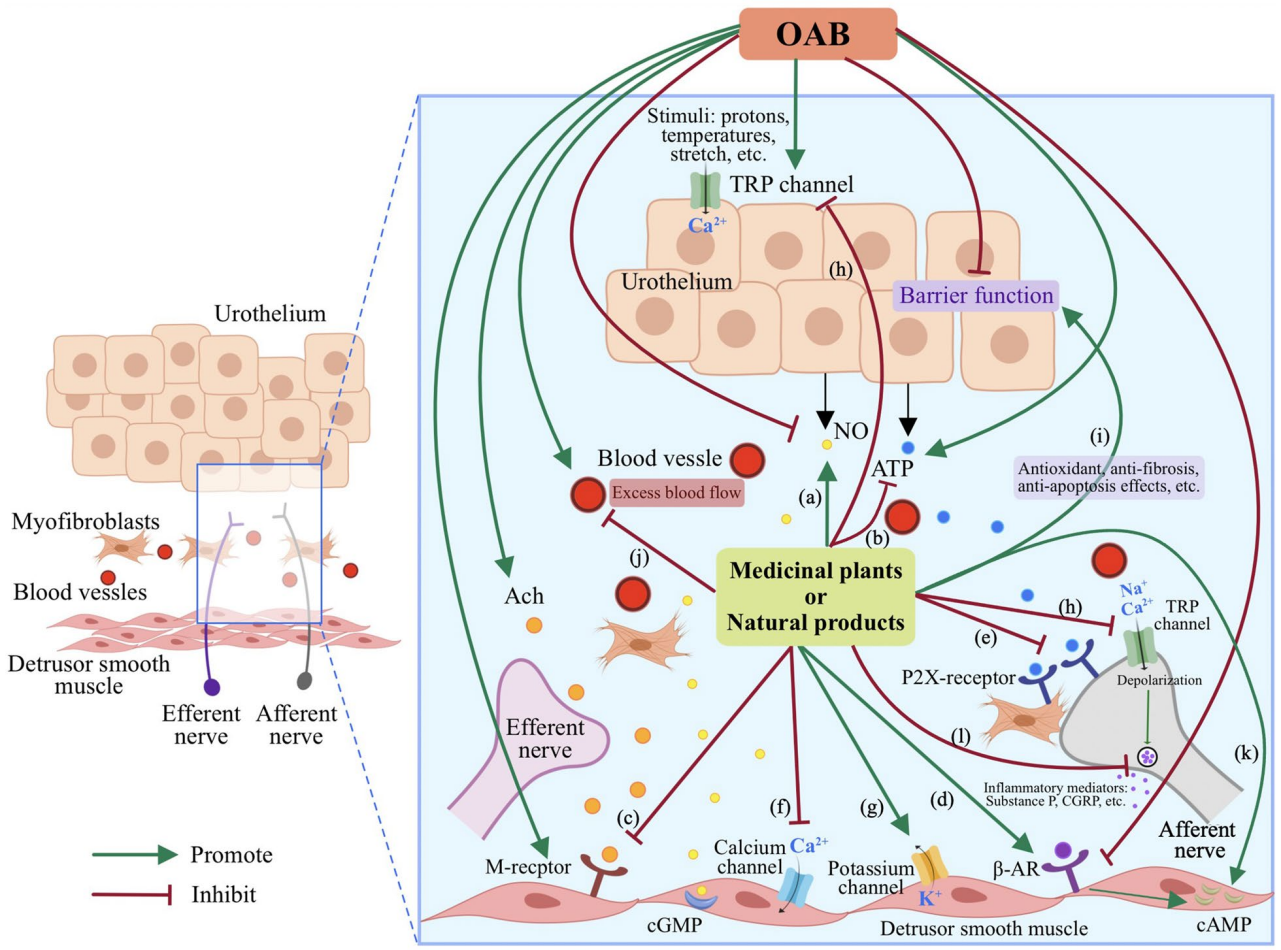
Illustration of possible intervention mechanisms of medicinal plants and natural products on OAB in the urinary bladder. **a** Promotion of the NO synthesis and release. **b** Reduction of the ATP synthesis and release. **c** Inhibition of the expression of the M receptors. **d** Up-regulation of the expression of the adrenergic receptors. **e** Down-regulation of the expression of P2X receptors. **f** Inhibition of transmembrane Ca^2+^ influx and stimulation of Ca^2+^ release from intracellular stores. **g** Activation of K^+^ channels. **h** Inhibition of TRP channels. **i** Improvement in the barrier function of urothelium. **j** Regulation of excess blood flow. **k** Increase in intracellular cAMP in the bladder smooth muscles. **l** Inhibition of the release of inflammatory mediators, such as Substance P, CGRP etc.

Literature reviews show a significant amount of research addressing the potential application of medicinal plants and natural products in OAB treatment [12, 34, 96]. A wide variety of medicinal plants have been employed to treat OAB in various regions and countries throughout different historical periods. Clinical evidence as well as preclinical in vitro and in vivo investigation have demonstrated that medicinal plants and/or their active ingreidents are efficacious for alleviating OAB. Additionally, herbs or medicinal plants are usually combined into therapeutic formulas in accordance with the theories of traditional medicine. TCM practitioners believe in “synergistic” or “emergence” effect when using herbal formulations, which are considered more effective and comprehensive in the treatment of diseases than single herbs alone. In this section, single-herb medications and herbal formulations for OAB treatments are reviewed and summarized in Tables 2 and 3 respectively. And the illustration of their possible interverntion mechanisms in the unrinary bladder is shown in Fig. 2.

### Single-herb medications for OAB treatment

#### *Alpinia oxyphylla*

*Alpinia oxyphylla* is known as “yì zhì (益智)” in Chinese. For centuries, the capsular fruit of this plant has been widely utilized in TCM to address specific symptoms of urinary incontinence, including frequency, urgency, and nocturia. Izalpinin, a flavonoid isolated from the fruit of *Alpinia oxyphylla*, was found to antagonized Carbachol-induced contractions concentration-dependently on rat bladder detrusor strips, which is due to its muscarinic receptor antagonistic action [168].

#### *Artemisia monosperma* (Wormwood)

This plant thrives extensively in the Arabian desert and has been deemed as an antispasmodic and anthelmintic in traditional medicine. It is also applied to treat hypertension. An isolated flavanone from *Artemisia monosperma*, 7-*O*-Methyleriodictyol, inhibited the amplitude of the phasic contractions in a dose-dependent fashion, and lowered the tone of ileum, uterus, and urinary bladder in rats. It also relaxed the phenylephrine-precontracted pulmonary artery and the acetylcholine-precontracted trachea [3].

#### *Artemisia vulgaris*

*Artemisia vulgaris* is widely distributed across natural habitats gloablly, spanning Asia, Europe, North and South America, as well as Africa [38]. It has been employed as a culinary spice in the food industry in different regions worldwide. For many centuries, *A. Vulgaris* has been utilized in traditional Chinese, Hindu, and European medicine to regulate the gastrointestinal system function and address a range of gynecological diseases [38]. In traditional system of medicine, this herb is considered a beneficial therapeutic agent in alleviating smooth muscle spasms [68]. In a recent research, it was found that *A. Vulgaris* extract inhibited carbachol-induced urinary bladder contractions via dual, anticholinergic and Ca^2+^ antagonist mechanisms, by blocking muscarinic receptors and influx of extracellular calcium [78]. This indicates its potential for treatment of bladder overactivity.

#### *Aspalathus linaeris*

*Aspalathus linearis*, commonly know as Rooibos (meaning “red bush”) is a leguminous shrub indigenous to the Cape Floristic Region of South Africa [7]. The species complex composes of several different growth forms, and among them the Red type has been cultivated for producing rooibos herbal tea. In the last decades, *A. linaeris* has become very popular for its antioxidant and medicinal attributes [122]. In traditional medicines, *A. linaeris* is generally accepted as a helpful therapeutic in relieving smooth muscle spasms [68]. In a study with rabbit urinary bladder strips, *A. linaeris* extract relaxed the bladder and inhibited carbachol-induced urinary bladder contractions via a dominant opening of ATP-sensitive potassium-channels and a weak blockade of calcium channels [78]. This reveals its medicinal usefulness in hyperactive bladder disorders.

#### *Astragalus membranaceus* (Huangqi)

*Astragalus membranaceus*, also called huáng qí (黄芪), it is an herb widely used in TCM and diet, and it has been widely studied in western medicine for various disease treatment. Astragalus polysaccharide (APS) is a type of water-soluble heteropolysaccharide deprived from the stems or dried roots of Huangqi [174]. Astragaloside IV (AS-IV), a small molecular saponin, is another major component from the aqueous extract of *Astragalus membranaceus* [170]. An in vivo study reported that both APS and AS-IV ameliorated urinary frequency on a CYP-induced OAB female mice model via modulation of urothelial wound healing, possibly via the increased expression of tight junction protein ZO-2 [32].

#### *Bletilla striata*

*Bletilla striata* is distributed widely in eastern Asian countries, including China, Japan, North Korea, and Myanmar. As widely used in TCM for thousands of years, its functions of hemostasis, detumescence, and improving one’s health have been recorded in Chinese Pharmacopeia (2015) [69]. Besides having been employed to treat hemoptysis, traumatic bleeding, chapped skin, swelling, and ulcer bleeding [157], it has also been used in TCM empirically to treat interstitial cystitis (IC). Study on zymosan‐induced cystitis in female rats [97] showed that treatment of *B. striata* extract solution decreased abdominal withdrawal reflex (AWR) scores and amplitude of bladder detrusor electromyogram (EMG). Furthermore, it demonstrated notable improvements in OAB by effectively prolonging the micturition interval and enhancing urine storage capacity. These results implied the possible efficacy of *B. striata* on treating OAB. However, more research on OAB models and patients is required for further validation.

#### *Bridelia ferruginea*

This tropical plant is native to Africa and has been utilized in African traditional medicine to treat intestinal and bladder ailments. Ethanolic extract of *B. ferruginea* leaves inhibited KCl-induced contractile response in rat urinary bladder smooth muscle. This effect might be ascribed to the blockade of purinergic neurotransmission [116]. It can be speculated that *B. ferruginea* leaf exact could be useful in bladder overactivity although further research is required to better evaluate its potential therapeutic application.

#### *Bryophyllum pinnatum*

*Bryophyllum pinnatum* is a succulent perennial plant originating in Madagascar, whose leaf press juice was shown to reduce the electrical- or carbachol-induced contractile response in porcine DSM [129]. The flavonoid fraction of this plant lowered the porcine detrusor contractility in dose- and time-dependent fashion [48]. In addition, results from a clinical trial with twenty postmenopausal women demonstrated that the leaf extract improved OAB symptoms and reduced micturition frequency after 8-week treatment [18].

#### *Camellia sinensis* (Green tea)

Green tea is a widely consumed healthy beverage around the world, known for its potent anti-inflammatory/antioxidant properties. Previous studies have identified these beneficial properties and attributed them to the presence of polyphenols in green tea. As the predominant catechin in green tea, epigallocatechin gallate (EGCG), is a well-established polyphenol flavonoid with renowned antioxidant activities. It was reported that EGCG restored ovariectomy (OVX)-induced bladder dysfunction dose-dependently through antioxidant, anti-fibrosis and anti-apoptosis effects on a surgical menopause-induced OAB rat model [72]. In addition, studies have revealed that EGCG can enhance bladder storage function and provide protection against interstitial fibrosis induced by Metabolic syndrome (MetS) and OVX in rats, potentially through the mitochondria and endoplasmic reticulum (ER) apoptosis pathways [89].

#### *Cananga odorata*

Ylang ylang essential oil is extracted from *Cananga odorata*, a medicinal plant grows in the north-east of Brazil. It possesses myorelaxant and antispasmodic properties, and it is used to relieve intestinal spasms as a smooth muscle relaxant [100]. In vivo and in vitro studies showed that ylang ylang essential oil inhibited urinary bladder contractile response induced by EFS and various agonists on rat and rabbit urinary bladders. The relaxing effect on bladders is thought to be mediated by c-AMP pathway [79].

#### *Cannabis sativa*

The plant *Cannabis sativa*, originated from Central Asia, has been used medicinally and recreationally, also as a source of textile fiber for thousands of years. It has been evaluated for its protective effect against LUTS and OAB caused by multiple sclerosis (MS). Clinical studies consistently revealed that standardized extracts derived from *Cannabis sativa*, such as Sativex, containing delta-9 tetrahydrocannabinol (THC) and cannabidiol (CBD), have been effective in reducing incontinence episodes in individuals with MS [24, 46, 149]. Later, a multicenter double-blind randomized clinical trial reported that Sativex reduced the number of nocturia episodes, voids per day and daytime voids, and improved Patient’s Global Impression of Change (PGIC) in patients with OAB due to MS [76]. It was found that extract enriched with cannabidiol inhibited cholinergic-mediated urinary bladder smooth muscle contraction in rats and human bladders. This effect is modulated by TRPV1 in rats but not in human. The inhibition was not observed in bladder contractions induced by potassium chloride (KCl), electrical field stimulation (EFS), or α,β-methylene adenosine triphosphate (α,β-MeATP) [26].

#### *Capsicum annuum* (Chili pepper)

*Capsicum annuum* fruit, usually termed as chili pepper, has been broadly used as food vegetables, natural colorants and flavoring ingredients, as well as a crude drug in many traditional medicine systems since ancient times [56]. Capsaicin is an active ingredient originated from chili peppers, found in the fleshy part of the pepper, rather than the seeds. Intravesical instillation of capsaicin can induce desensitization of TRPV1 receptors on afferent nerves, leading to a reduction in neural firing, and inhibition of the micturition reflex. As a TRPV1 channel agonist, capsaicin attaches to and activate the vanilloid receptors, leading to calcium entry through the neuronal membrane, and then resulting in desensitization of the nerve, hence, analgesia. Also, binding of the receptor causes a decrease in substance P, a major pain neurotransmitter [55]. But capsaicin often causes local irritation and edema. Attempts to translate these desensitization activity have been used to the bladder control. In a meta-analysis comprising eight open-label and two placebo-controlled clinical trials involving 200 patients with lower urinary tract disorders, intravesical capsaicin treatment for neurogenic hyperreflexic bladder demonstrated clinical or urodynamic symptom improvement in 84.3% of the patients. However, side effects appear during or immediately after instillation [36]. Application of capsaicin in overactive bladder is very much limited.

#### *Citrus depressa*

*Citrus depressa* (shekwasha), is a kind of citrus fruit produced in southern part of Japan. A study by Ito et al. discovered that nobiletin, a polymethoxy flavonoid present in shekwasha abundantly, significantly improved hyperactive urodynamic symptoms in CYP-induced cystitis rats by decreasing micturition frequency. The beneficial effect may be partly attributed to the potency of raising intracellular cAMP level in the bladder smooth muscles [67].

#### *Cucurbita pepo* (Pumpkin)

Pumpkin seed oil obtained from *Cucurbita pepo* presents a strong antioxidant activity and valuable nutritional benefits. It is useful for treating a range of diseases, such as benign prostatic hyperplasia (BPH), urinary disorders, hypertension, hyperlipidemia, diabetes, and cancer [115]. In an in vivo urodynamics study on rabbits, it was reported that a preparation of pumpkin seed oil reduced bladder pressure, improved bladder compliance, and reduced urethral pressure in rabbits [173]. In a study involving 45 patients with symptoms of OAB, pumpkin seed oil was investigated and found to significantly decrease the Overactive Bladder Symptom Score (OABSS) without any observed adverse reactions over a 12-week treatment period [113]. In a recent single-blind randomized clinical trial involving 73 subjects with BPH aged ≥ 50 years, pumpkin seed oil decreased International Prostate Symptom Scores (IPSS) and improved QOL in patients, with BPH symptoms alleviated and no side effects [169].

#### *Cyclotrichium niveum*

It is a Turkish flora widely used as tea. The essential oil of *C. niveum* relaxed of carbachol precontracted rabbit bladder strips, which showed the antispasmodic activity of *C. niveum*. The essential oil also exhibited radical scavenging effects [29]. Results showed its possible effects on reducing OAB symptoms, however, further studies must be undertaken to verify this speculation.

#### *Euphorbia resinifera* (Resin spurge)

A cactus-like plant commonly found in Morocco, *Euphorbia resinifera* contains the naturally occurring constitutent resiniferatoxin (RTX), which is a potent functional analog of capsaicin and is a potent TRPV1 agonist. By selectively binding to the TRPV1 receptor, intravesical RTX effectively obstructs the afferent nerves responsible for transmitting pain sensations to the brain. A meta-analysis of seven trials involving 355 patients diagnosed with either interstitial cystitis (IC) or DO reported that RTX resulted in an increase in maximum cystometric capacity (MCC) in patients with DO and provided relief from bladder pain in patients with either IC or DO. Nevertheless, no significant improvement in frequency, nocturia, incontinence or first detrusor contraction (FDC) was observed [52]. On the other hand, other study has presented an opposite result of higer pain sensation, lower bladder capacity, increased urinary frequency and nociceptive behaviors (such as licking and freezing) after instillation of RTX into rat bladders [126]. RTX is rarely used clinically due to its inconsistency in efficacy, difficulties in delivery and acute pain.

#### *Galium aparine*

It is found in North America, and it’s an annual herbaceous plant that grows on roadsides, pastures and uncultivated places. Due to presence of hooks, this plant can’t be consumed raw and is usually used in teas. It has been employed in cystitis traditionally although it hasn’t been demonstrated pharmacologically [25]. Its possible efficacy of treatment OAB might be worth exploring with scientific studies.

#### *Ganoderma lucidum*

Medicinal benefits of the fungi *Ganoderma lucidum* has been widely recognized for thousands of years in East Asia (mainly China, Japan and Korea). Its fruiting body is called “Reishi” in Japan and “líng zhī (灵芝)” in China. Traditionally, it is known as “longevity-promoting-tonic” and has been used in China in the Qi replenishment, mind relaxation, as well as easing the cough and asthma [5]. And in the modern medicine systems, it has been applied to cure different ailments such as hepatitis, hypertension, hypercholesterolemia, diabetes and various cancers [127]. In a randomized, double-blind, placebo-controlled and dose-ranging clinical study on 50 male volunteers (≥ 50 years old), the extract of *Ganoderma lucidum* demonstrated an improvement in IPSS among men with LUTS with no major adverse effects reported [114]. Through an in vivo rabbit model of ischemia/reperfusion (I/R), it was observed that *Ganoderma lucidum* exhibited antioxidant effects, effectively mitigating the detrimental impact of I/R-induced oxidative stress on bladder compliance and contractile responses [90].

#### *Glycine max* (Soybean)

*Glycine max* is a species of legume native to East Asia, also termed as the soybean or soya bean [152]. On isolated strips of rabbit detrusor, genistein, a major dietary phytoestrogen from soybean exhibiting tyrosine kinase inhibitory activity, were shown to induce relaxation in detrusor muscle contracted by the muscarinic receptor agonist bethanechol (BE) and the purinergic P2X receptor agonist α, β-methylene ATP (α, β-MeATP). Genistein exhibited a selective reduction in peak contractions induced by α, β-MeATP and steady-state contractions induced by BE, possibly through inhibition of voltage operated Ca^2+^ channels (VOCCs) [120]. A cross-sectional study involving a substantial sample of 2000 elderly Chinese men has shown that dietary intake of soy isoflavones was linked to a lower risk of LUTS [155]. Later in another study, two soy isoflavones genistein and daidzein were reported to dose-dependently decrease detrusor contractions induced by EFS probably via activation of large and small conductance K^+^(Ca) channels [147].

#### *Hippophae rhamnoides* (Searberry)

Seaberry or sea buckthorn obtained from *Hippophae rhamnoides*, which grows in Northern Europe, Western Asia, China and Canada, has shown inhibitory effect on carbachol induced contractions in rat bladder strips and on TGF-β-induced constrictions in human bladder smooth muscle cells. Triterpenoids and flavonoid glycosides in seaberry which include 3-O-coumaroyl 2,23-dihydroxy oleanolic acid, ursolic acid, uvaol, pomolic acid, oleanolic aldehyde and isorhamnetin 7-O-rhamnoside, might contribute to this protective activity. Among them, ursolic acid (1–100 μM) and isorhamnetin 7-O-rhamunoside (10 μM) remarkably inhibited carbacol-induced bladder constraction [132]. In a clinical trial of seaberry extract supplementation in Japanese men and women with mild urinary dysfunction, several emotional parameters associated with urinary dysfunction were improved, suggesting that it may be beneficial for relieving moderate urinary symptoms [140].

#### *Hypericum perforatum*

*Hypericum perforatum* is a herbaceous perennial plant originating in Asia and Europe, and it has been introduced into the United States [16]. St John’s wort (SJW), obtained from the leaves and flowering tops of *H. perforatum*, is a well-known successful herbal antidepressant. The investigation on its effect on bladder contractions showed that it inhibited EFS-contractile response in isolated rat bladder smooth muscle strips. Opioid receptors might be involved in inhibitory activity on excitatory transmission of SJW [27].

#### *Perilla frutescens*

*Perilla frutescens* is an aromatic plant exhibits a wide distribution in East Asian countries, such as China, Japan, Korea, and Vietnam. It has been cultivated as an edible cropand used in TCM since ancient times [58]. Perilla leaves have served in preparation of vegetable curries, chutneys and pickles. Perilla has also been applied in medical and pharmacological terms, for its anti-oxidant, anti-inflammatory and anti-allergy activities. An in vivo study [84] showed that 2-week perilla extract treatment notably enhanced the micturition interval in female spontaneously hypertensive rats (SHRs) without significantly affecting maximal pressure, suggesting that perilla improves frequent urination, without suppressing contraction of the detrusor muscle. In comparison to the control group, the perilla group displayed a reduction in expression of nerve growth factor (NGF), tumor necrosis factor- α (TNF-α), interleukin-1β (IL-1β) and TRPV1, as well as an increased level of uroplakin 3A (UPK3A). Thin or defective urothelium was detected in the control group, while the perilla group displayed nearly complete preservation of the urothelial integrity. Additionally, being the main components of perilla extract, perillaldehyde and perillic acid, inhibited the induction of NGF and TNF-α by IL-1β in vitro [84]. These results imply effects of perilla on OAB is likely be modulated, at least partly, by improvement of the urothelial presence and by the anti-inflammatory activities of perilla.

#### *Peucedanum japonicum*

The root of *Peucedanum japonicum* is traditionally used to treat inflammatory diseases in southern parts of Japan. Preclinical studies showed that extract from *Peucedanum japonicum* inhibited agonist-induced rabbit bladder contractile response and improved urodynamic symptoms in hyperactive rat bladders with a reduction of micturition frequency, while its active ingredient, isosamidin, demonstrated the ability to reduce or mitigate phenylephrine‐activated contractions in isolated human prostate tissue strips [65, 139]. On the other hand, clinical effects of the extract were investigated in male patients with LUTS. It improved urodynamic parameters and subjective symptom scores without reported adverse drug reactions [74]. These investigations suggest its possible therapeutic application in OAB treatment.

#### *Potentilla chinensis*

*Potentilla chinensis* is a perennial herb can be found extensively across Korea, Japan and China. In Korea, it has been traditionally utilized as a medicinal remedy to treat inflammation, myalgia, scabies, and dysentery [53, 143]. Wróbel et al. found that the aqueous extract of *P. chinensis* alleviated retinyl acetate (RA)-induced DO in rats, and the mechanisms may involve the extract’s ability to inhibit the release of transmitters from both afferent and efferent fibers that innervate the urinary bladder, and its influence on the exocytotic process depending on SNARE (soluble N-ethylmaleimide-sensitive-factor attachment protein receptor) protein activity [156]. More recently, aqueous extract of *P. chinensis* (PCE) was found to attenuate DO in rats suffering from cyclophosphamide (CYP)-induced hemorrhagic cystitis. Considering PCE contains an abundance of antioxidants, the protective activities of PCE appear to be related to preventing oxidative stress-dependent dysfunction of the urinary bladder [73].

#### *Puerariae lobatae* (Gegen)

The Chinese herb Gegen (gé gēn 葛根) is the dried root of *Puerariae lobatae* (wild), and has been traditionally employed to treat a wide range of symptoms including diarrhea, acute dysentery, deafness and cardiovascular diseases. In an ex vivo experiment on isolated rat bladder strips, Gegen water extract caused relaxation of detrusor muscle in urothelium-independent fashion, and it acted synergistically with the water extract of *Salviae Miltiorrhizae* Radix (Danshen) [92]. Later, an in vivo study [175] showed that water extract of Gegen reduced carbachol-induced tonic contractions in male spontaneously hypertensive rats (SHR) but did not alter the amplitude of phasic contractions. Combination of Gegen and darifenacin exhibited synergetic effect on inhibition of electric field stimulation (EFS)-induced contractions. Gegen improved DO through neurogenic and anti-muscarinic action, more specifically, on M_3_ receptor.

#### *Rhois aromatica*

*Rhois aromatica* is originated from Northern America. Its extract was shown to inhibit carbachol and KCl-induced contractile response in rat and human bladders. This reduction of contractile response involved direct antagonistic effect on muscarinic receptors and receptor-independent mechanisms [21].

#### *Salvia cinnabarina*

It is an American species of the genus of *Salvia*, which is used in folk medicine for its various bioactivities. It has been shown that a secoisopimarane diterpenoid from *Salvia cinnabarina*, 3, 4-secoisopimar-4(18), 7, 15-triene-3-oic acid significantly inhibited EFS-contratile response in isolated rat urinary bladder in a dose-dependent manner, with a mechanism related to, at least partly, NO production [28].

#### *Serenoa repens* (Saw palmetto)

*Serenoa repens*, commonly accepted as saw palmetto, is a small, low-growing, dwarf-palm tree endemic to the south eastern America and West Indies [4]. Traditionally, the berries were used as a staple food and medicine. It has been used to treat diarrhoea and stomach ache, and served as a diuretic and sexual tonic [154]. Several clinical studies showed that administration of saw palmetto (*Serenoa repens*) extract improved the IPSS in patients with LUTS and alleviated urodynamic symptoms including urination frequency and nocturia [41, 51, 74]. In a clinical research encompassed 591 patients with inflammation assosciated chronic benign prostate conditions, saw palmetto extract (SPE) mitigated bladder voiding, LUTS as well as erectile function, and improved IPSS and National Institute of Health—Chronic Prostatitis Symptom Index (NIH-CPSI) [51]. Another study on 20 male patients (≥ 50 years old) with untreated LUTS and no serious complications showed that 4-week SPE administration improved IPSS-QOL score, nocturia, and OABSS-2 [74]. The efficacy and safety of SPE (12-week treatment) were studied in 76 adult women with urinary symptoms and the results showed SPE significantly alleviated daytime frequency and nocturia with high safety [162]. In line with this, preclinical study on obese male Wistar rats showed SPE improved smooth muscle fiber structure and reduced cell proliferation in the bladder, indicating its potential beneficial effects on LUTS [41]. Several mechanisms of action have been proposed for SPE, which include anti-inflammatory/anti-oedematous effect, anti-androgenic effect, prolactin signal mediation, and anti-proliferative action via inhibiting growth factors [50].

#### *Silybum marianum*

*Silybum marianum,* known as milk thistle, is indigenous to Northern Africa, Southern Europe, Southern Russia and Anatolia and also distributed in South Australia, North and South America [102]. For thousands of years, it has been used as a remedy for a range of liver dysfunctions and gallbladder disorders [19]. Silymarin, a mixture of flavonolignans obtained from *S. marianum*, is the active component of this herb. It includes mainly silybin A, silybin B, isosilybin A, isosilybin B and other flavonolignants such as silychristin, neosilyhermin, silyhermin and silydianin, which are predominantly found in the fruits and seeds of the plant compared to other parts [75]. It was shown to reduce cyclophosphamide-induced enhanced contractile response in CYP-induced cystitis rat model, suggesting its possible application in treating bladder overactivity in cystitis. This action may be associated with its antioxidant and anti-inflammatory activities [40].

#### *Solanum lycopersicum* (Saladette tomato)

Saladette tomato, scientifically referred to as *Solanum lycopersicum*, holds significant importance as one of the key vegetable plants globally. It orginated in western South America, with its domestication considered to have taken place in Central America [83]. In an in vivo study on high-carbohydrate diet induced obese Wistar rats (male), lipidic extract of saladette tomato reduced hyperplasia and contractility, improved fiber structure of smooth muscle and decreased cell proliferation in the bladder, revealing its potential protective effects on LUTS and OAB [41].

#### *Solidaginis virgaurea*

It is native to middle Europe, and it is the most frequently used plant for extraction to produce preparations in phytotherapy to treat bladder dysfunction including the OAB syndrome. Extract from the *Solidaginis virgaurea* showed inhibitory action on carbachol and KCl-induced contractile response in rat and human bladders, suggesting its possible efficacy on treating OAB. Additionally, direct antagonistic effect on muscarinic receptors and receptor-independent mechanisms of *Solidaginis virgaurea* are related to the reduced contractility [21].

#### *Uncariae Ramulus Cum Uncis*

*Uncariae Ramulus Cum Uncis* (Gambir Plant or gōu ténɡ 钩藤 in Chinese) is a herbal medicine that has enjoyed broad usage in China and Japan for thousands of years. It mainly grows in tropical regions, such as Southeast Asia, Southeast America and Africa [171]. Rhynchophylline, as the main active component of *Uncariae Ramulus Cum Uncis*, has been discovered to inhibit the constriction of isolated rat urinary bladder strips ex vivo, and improved urodynamic parameters in rats in vivo, through activating calcium-activated potassium channels and blocking L-type calcium channels [70, 71]. Rhynchophylline inhibited the intracellular-calcium-induced contractions of rat bladder strips at a low concentration (10 μmol/L), whereas it inhibited both intracellular- and extracellular-calcium-induced contractions at a high concentration (20 μmol/L) [70]. Based on these findings, it can be inferred that Rhynchophyllineplays has the potential to serve as an alternative therapeutic agent for OAB treatment.

#### *Vaccinium corymbosum* (Blueberry)

Blueberries (*Vaccinium corymbosum*) have a rich historical background of being utilized both as a food source and for medicinal purposes in Europe and North America. Nowadays they are widely consumed as a health food worldwide. They are commonly considered as one of the most abundant sources of antioxidant phytonutrients among the fresh fruits and vegetables that have been researched [134]. A study by Miyazaki et al. reported that blueberries successfully averted the onset of bladder dysfunction resulting from BOO in rats via antioxidative effects and inhibiting bladder remodeling, and that these two effects might act synergistically to exert a preventive activity [108].

#### *Vanilla planifolia*

It is a precious orchid originating in Mexico and Central America, and has been cultivated in various tropical regions worldwide for production of natural vanilla flavor [22]. The scent of vanilla has been applied to treat sleep disorders thanks to its relaxing effect. It has been reported that vanilla oil decreased serum catecholamine (adrenaline, noradrenaline and dopamine) levels, increased intervals between bladder contractions, decreased urination frequency in rats in a sleep-like state induced by light urethane anesthesia, indicating that it may reduce nocturia by reducing sympathetic activity [138].

#### *Vitis vinifera* (Grape)

*Vitis vinifera* is a climbing vine cultivated worldwide, and the largest fruit crop in the world [131]. It has served as a nutritional supplement or food colouring additive in the food industry [42]. Thanks to its antibacterial activity, *V. vinifera* has been proposed as an alternative to chemical preservatives [118]. The fruits of *V. vinifera*, namely grapes, have extensive application in the production of juices, wines, and raisins [133]. In the pharmaceutical industry, *V. vinifera* serves as a valuable source of raw materials renowned for their antioxidative, hepatoprotective, cardioprotective, anticancer, antiviral and antibacterial effects. The potential utilization of *V. vinifera* or the derived active compounds as environmentally friendly agents with antibacterial or anticancer properties have been also reported [13, 87]. In addition, the production of cosmetics with grape extract is specifically popular worldwide nowadays. Raw materials deprived from *V. vinifera* have gained significant recognition and widespread use in cosmetics, especailly for their antioxidant, anti-ageing, UV-protection and skin-whitening activities. Furthermore, the safety of *V. vinifera* has been well-established, further enhancing its appeal for cosmetic applications. [131].

The protective effects of grape suspension and resveratrol against increased contractions and voiding frequency have been confirmed in multiple animal studies [6, 44, 167]. Resveratrol is considered to be the basic active ingredient responsible for the antioxidant properties of grapes [60]. In studies carried out by Francis et al. on H_2_O_2_-induced oxidative stress in rabbit urinary model, it was observed that whole grape suspension produced a higher reduction of citrate synthase activity in muscle and mucosa compared to resveratrol. Whole grape suspension also showed higher protective effect than resveratrol against choline acetyltransferase activity, which is responsible for the synthesis of Acethylcholine and finally causes bladder smooth muscle contractile response. It was concluded that whole grape suspension were more effective in ameliorating oxidative stress than resveratrol, suggesting combinational benefits of the active components [44, 45]. In addition, resveratrol was reported to improve overactive bladder via downregulation of the protein expression level of SCF, c-Kit and p-AKT in the bladder of rats with chronic prostatitis (CP), and the combination of resveratrol and solifenacin strengthened the improvement in overactive bladder, indicating potential pharmacological synergy as a theraputic strategy for CP patients [167].

#### *Zea mays* (Cornsilk)

Cornsilk is obtained from the female flower of corn (*Zea mays*). Its application in cystitis that involves OAB symptoms has been reported [30, 34]. However, there are no pharmacological or clinical studies that have been carried out regarding this effect. It might be worth exploring.

**Fig. 3:**
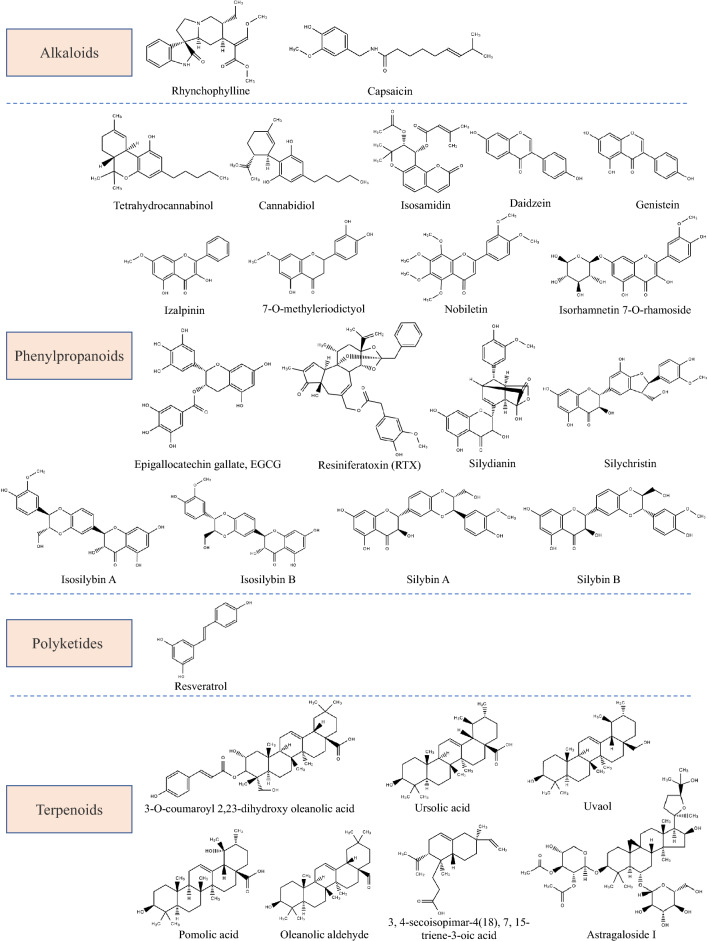
Structures of phytochemicals used for treating OAB

Natural products are often classified into four groups according to their biosynthetic origins and major structural features: alkaloids, phenylpropanoids, polyketides, and terpenoids[136]. From the herbs mentioned, a number of phytochemicals have been isolated and identified for their pharmacological activity against OAB. Summary and classification of these compounds are shown in Fig. 3.

### Herbal formulations for OAB treatment

#### TCM formulations

##### Bu-Zhong-Yi-Qi-Tang

Bu-Zhong-Yi-Qi-Tang (BZYQT) is an herbal formula described in the Pi Wei Lun (脾胃论, Treatise on Spleen and Stomach), a medical text dating back to 1249 AD. This formulat primarily targets spleen and stomach deficiencies, which are are considered as the origin of various health disorders. Translated typically as “the decoction for strengthening the center and enhancing qi,” the name Bu-Zhong-Yi-Qi-Tang encapsulates the essence of this herbal formula (Kim et al., 2017b). This formula consists of ten herbs, including *Radix Astragali* (huáng qí 黄芪), *Rhizoma Atractylodis* (bái zhú 白术), *Radix Panacis Ginseng* (rén shēn 人参), *Radix Angelicae* (dāng guī 当归), *Radix Bupleuri* (chái hú 柴胡), *Fructus Zizyphi* (dà zǎo 大枣), *Aurantii Nobilis Pericarpium* (chén pí 陈皮), *Radix Glycyrrhizae* (gān cǎo 甘草), *Rhizoma Cimicifugae* (shēng má 升麻), *Rhizoma Zingiberis* (shēng jiāng 生姜). In a few clinical studies carried out in China, BZYQT showed significant efficacy in treating OAB, both alone [91] and in combination of Sang-Piao-Xiao-San [95] or propiverine [33]. BZYQT is believed to stimulate the flow of Qi, which serves as essential energy for the body to nourish the internal organs and maintain physical activities.

##### Ba-Wei-Di-Huang-Wan (Hachi-mi-jio-gan)

Ba-Wei-Di-Huang-Wan (BWDHW), also known as Hachi-mi-jio-gan in Japanese, is traditionally applied to warm the kidney yang and alleviating frequent urination. It is one of the most frequently used TCM prescription for treating abnormal thirst, polyuria, polydipsia, and urinary frequency with diabetes-like symptoms. The BWDHW regimen was created by the renowned Dr. Zhongjing Zhang (张仲景) over 1800 years ago. This herbal mixture contains eight ingredients, including *Rehmanniae radix* (dì huáng 地黄), *Cornus officinalis* (shān zhū yú 山茱萸), *Dioscoreae rhizoma* (shān yào 山药), *Alismatis rhizoma* (zé xiè 泽泻), *Porica cocos* (fú líng 茯苓), *Moutan radicis cortex* (mǔ dān pí 牡丹皮), *Cinnamomi cortex* (guì pí 桂皮), and heat-processed *Aconiti radix* (fù zǐ 附子). Nowadays, the granules of BWDWH manufactured by modern pharmaceutical companies are widely used by practitioners in Asia.

BWDHW exhibits potential in treatment for bladder overactivity associated with diabetes and metabolic syndrome in rats. In streptozotocin-induced diabetic rats, Tong et al. investigated the effects of BWDHW on the cholinergic function of the bladder and reported that BWDHW lowered the plasma glucose and reversed the hyper-contractility in the bladder. Results from this study indicated that BWDHW inhibits M_2_ receptors overexpression and attenuats bladder overactivity [144]. Hachi-mi-jio-gan extract (HARNCARE^®^, HE), a galenical made from BWDHW, was reported to inhibit acetylcholine-induced contraction of rat bladder strips and exert significant binding activity to muscarinic receptors, 1,4-dihydropyridine (DHP) receptors and purinergic receptors in the bladder [66]. Moreover, the effects of THC-002, an ethanol extraction of BWDHW (HARNCARE®), were tested on SHRs and it was reported that THC-002 effectively down-regulates the expression of tachykinins and P2X_3_ and TRPV1 receptors in the bladder, thus inhibiting adenosine triphosphate (ATP)-induced DO [63]. Later on, Lee et al. demonstrated that BWDHW treatment ameliorated CYP-induced ongoing bladder hyperactivity by suppressing protein overexpression of mucosal P2X_2_, P2X_3_, M_2_, and M_3_ receptor, as well as detrusor M_2_ and M_3_ receptor in rat bladders exposed to CYP treatment. In addition, BWDHW pretreatment reduced acidic ATP solution-induced bladder hyperactivity in rats by preventing hypersensitization of TRPV1 receptor in bladder mucosa activated by acidic stimulation and supressing overexpression of P2X_3_ receptor in naïve bladder mucosa [88]. Furthermore, Tsai et al. demonstrated that BWDHW, through its active ingredient loganin, alleviates bladder overactivity as well as modulates substance P (SP)-induced oxidative injury and inflammation by inhibiting SP/neurokinin-1 receptor and nuclear factor kappa B/intercellular adhesion molecule 1 (NF-kB/ICAM-1) signaling pathways [145].

##### Fangjihuangqi Tang

Fangjihuangqi Tang (FHT) is composed of four herbs: *Radix Stephaniae Tetrandrae* (fáng jǐ 防己) 12 g, *Radix Astragali Mongolici* (huáng qí 黄芪) 15 g, *Rhizoma Atractylodis Macrocephalae* (bái zhú 白术) 9 g, and *Radix Glycyrrhizae* (gān cǎo 甘草) 6 g. It was first described in the ancient Chinese medical classic Jin Gui Yao Lue (金贵要略, Synopsis of Golden Chamber), and has been one of the most common TCM prescription to treat dysuria and edema for more than 1800 years. A study on benign prostatic hyperplasia (BPH) rats showed that 4-week FHT treatment could alleviate symptoms of lower urinary tract dysfunction by modulating smooth muscles in the bladder and urethra instead of lowering the volume of the enlarged prostate itself. It was speculated that one of the mechanisms of FHT might be related to active ingredients with calcium channel blocking effects and thus the constraint of the internal flow of Ca^2+^ [31].

##### Ji‐Sheng‐Shen‐Qi‐Wan (Gosha-jinki-gan)

Ji-Sheng-Shen-Qi-Wan (JSSQW), also called Gosha-jinki-gan, has gained wide application as a treatment for patients suffering from LUTS, diabetic neuropathy, and nocturia in China and Japan. Traditionally, it is used to treat water accumulation caused by kidney yang deficiency. It was first recorded in the Southern Song Dynasty (AD 1127–1279). Containing 10 ingredients, JSSQW is a modification of BWDHW with the addition of *Plantaginis semen* (chē qián zǐ 车前子) and *Achyranthis radix* (niú xī 牛膝).

In basic research, JSSQW was found to improve bladder hyperactivity induced by intravesical installation of acetic acid (AA) through inhibition of resiniferatoxin (RTX)-sensitive bladder afferent neurons in rats [172]. In a rat model of AA induced OAB, Gosha-jinki-gan-fed rats exhibited longer intercontractile intervals and smaller contraction amplitudes in cystometry, and also lower dopamine and serotonin levels in plasma, compared with untreated controls. These results imply that JSSQW may have an impact on both the afferent and efferent micturition reflex and may exert central effects on micturition mechanisms. By regulating the balance of sympathetic and parasympathetic nervous systems to maintain a lower level of activity, it effectively restrains bladder overactivity [112]. Moreover, a later study demonstrated that pretreatment with JSSQW may prevent the bladder urothelium from overexpressing tachykinins (including neurokinin A, neurokinin B and substance P) and TRPV1 and P2X_3_ receptors in rats subjected to intravesical AA stimulation. Besides, the authors further indicated that JSSQW may reduced expression of transmitter proteins and sensory receptors, without damaging nerve fibers [62]. A clinical trial reported that patients with nocturia exhibited decrease in both nocturia episodes and the IPSS after 12-week JSSQW treatment [161]. Thus, as addressed in literature, JSSQW mixtures show potential in offering alternative therapeutic option for OAB.

##### Modified Ojayeonjonghwan (Wuzi Yanzong Wan)

Ojayeonjonghwan (Wuzi Yanzong Wan) consists of five different fruits and seeds obtained from five types of berries: *Psyllium, Cuscuta chinensis Lam*, *Lycium chinense Miller*, *Rubus coreanus Miquel*, and *Schisandra chinensis Baillon*. A new berry mixture formula was developed in South Korea, and it is a modification of Ojayeonjonghwan with Psyllium replaced by *Cornus officinalis Sieb*. Bae, Lee et al. used a partial BOO animal model to demonstrate that the modified Ojayeonjonghwan (MO) exhibited similar pharmacologic activities to solifenacin in controlling DO induced by BOO via anti-inflammatory effect and anti-oxidant effects, as well as the increase of the NO pathway [15].

##### Sang-Piao-Xiao-San (Mantis Formula)

Sang-Piao-Xiao-San (Mantis Formula, 桑螵蛸散, SPXS) was first described in Ben Cao Yan Yi (本草衍义, Augmented Materia Medica) in the Northern Song Dynasty (AD 960–1127) for its effectiveness in addressing conditions characterized by heart and kidney deficiency, such as urinary incontinence accompanied by spermatorrhea. It is composed of *Mantidis ootheca* (sānɡ piāo xiāo 桑螵蛸), *Radix Polygalae* (yuǎn zhì 远志), *Rhizoma Acori Tatarinowii* (shí chāng pú 石菖蒲), *Fossilia Ossis Mastodi* (Dragon’s Bone, lóng gǔ 龙骨), *Radix Panacis Ginseng* (rén shēn 人参), *Radix Angelicae* (dāng guī 当归), and *Carapax Testudinis et Plastrum.* Sānɡ piāo xiāo means “mantis eggs in a foamy pouch”. *Mantidis ootheca* is the main herb in this formula. It has been discovered to induce relaxation of vascular smooth muscle via endothelium-dependent activation of PI3K/AKT-mediated NO-cyclic guanosine 3′,5′-monophosphate (cGMP)-protein kinase G (PKG) signaling pathway, upregulating NO production, and via possible involvement of K + channel [80]. A clinical study in patients with OAB showed co-treatment of SPXS and BZYQT decreases urinary frequency, urgency and urge incontinence, and enhances the voided volume and patient QOL [95]. Lu et al., studied the effects of SPXS and solifenacin in patients with OAB after menopause. They reported that SPXS combined with solifenacin improved TCM symptom scores, as well as the scores of urination, nocturnal urination, urgency of urination and urgency incontinence compared to treatment with solifenacin alone. It improved bladder compliance, maximum urinary flow rate and maximum bladder capacity, the initial urine volume (VFD), and also lower NGF and NGF/Cr level to a greater extent than using solifenacin alone.

##### Suo-Quan-Wan

Suo-Quan-Wan (SQW) is a classical phytotherapeutic formula in TCM consisting of three herbs: *Alpinia oxyphylla Miq* (yì zhì rén 益智仁), *Lindera radix* (wū yào 乌药), and *Dioscorea rhizoma* (shān yào 山药). SQW was first recorded in the Southern Song Dynasty (AD 1127–1279) and has been frequently applied in relieving nocturnal enuresis and frequent urination. Lai et al*.* reported that SQW reduced TRPV1 receptors expression in rat bladders and slowed down the advancement of bladder overactivity in a rat model of partial BOO [85]. In a later study with TRPV1 knockout mice, they provided additional evidence that SQW enhances bladder function by regulating TRPV1 receptors [86]. In another study by Xu et al., SQW was found to enhance bladder control, storage capacity and contraction ability in aging mice by increasing the sensitivity and expression of β_3_-adrenoceptor (β_3_-AR) [160]. Recent research discovered SQW’s protective effects against OAB and bladder dysfunction in diabetic mice. SQW treatment remarkably improved urodynamic urination with reduced non-voiding contraction (NVC) frequency, maximum bladder capacity (MBC), residual volume (RV), and bladder compliance (BC), and enhanced voided efficiency (VE). It also attenuated thickened bladder wall in diabetic mice, decreased DSM strips contraction response for stimuli including α, β-methylene ATP and carbachol. The mechanism involves upregulating motor protein myosin Va and transporter protein SLC17A9 in the bladder [151].

##### Wenglitong capsule

Wenglitong capsule (WLT) is a medicinal preparation that contains 11 herbal ingredients: *Semen Coicis * (Ma‐yuen jobstears seed, yì yǐ rén 薏苡仁), *Bulbus Fritillariae Thunbergii * (Chekiang fritillary bulb, zhè bèi mǔ 浙贝母), *Caulis Clematidis Armandii* (Armand clematis stem, chuān mù tōng 川木通), *Fructus Gardeniae* (Cape jasmine, zhī zǐ 栀子), *Flos Lonicerae Japonicae * (Japanese honeysuckle flower bud, jīn yín huā 金银花), *Flos Inulae Japonicae * (Japanese inula flower, xuán fù huā 旋覆花), *Lycopi Herba * (Hirsute bugleweed herb, zé lán 泽兰), *Verdigris* (tóng lǜ 铜绿), *Radix Glycyrrhizae * (Liquorice root, gān cǎo 甘草), *Radix Astragali Mongolici* (huáng qí 黄芪) and *Rheum officinale* (dà huáng 大黄). In a clinical trial on 182 female OAB patients, treatment with WLT reduced urgency incontinence, urinary frequency and significantly improved OAB symptoms, while exhibiting a slower onset and lower effectiveness compared to tolterodine, the treatment presented a favorable profile with fewer adverse effects. In addition, The combined administration of WLT and tolterodine demonstrated greater effectiveness in alleviating symptoms of OAB compared to using tolterodine alone [159].

#### Non-TCM formulations

##### Choreito (CRT)

As a traditional Japanese (Kampo) medicine, Choreito (CRT) finds extensive application in Japan to treat OAB and other lower urinary tract symptoms. CRT consists of five ingredients including aluminum silicate hydrate with silicon dioxide, *Alisma* rhizome, *Polyporus* sclerotium, *Poria* sclerotium, and donkey glue. In rats, CRT demonstrated the ability to reduce DO induced by intravesical AA instillation, which appears to be mediated via alleviating urothelial damage and regulating excess blood flow [146].

##### Eviprostat

The phytotherapeutic agent Eviprostat is a herbal remedy, consists of a mixture of whole plant extract deprived from several plants including *Chimaphila umbellate* (umbellate wintergreen), *Populus tremula* (aspen), *Pulsatilla pratensis* (small pasque flower) and *Equisetum arvense* (horsetail). This combination is blended with wheat-germ oil. With a history of over 50 years of prescription in Germany and Japan, it is one of the most extensively utilized phytotherapeutics to treat LUTS in BPH. And it is well known to exhibit antioxidant and anti-inflammatory activities, which have been investigated and validated in multiple studies. It was reported that Eviprostat was found to reduce the levels of 8-hydroxy-2’ –deoxyguanosine (8-OHDG), a urinary marker of oxidative stress, in both a rabbit model of surgically induced partial BOO and patients with LUTS related to BPH [104]. In another animal study on a rat model of bladder overdistension and emptying (OE), Eviprostat reduced inflammation and oxidative stress in OE rat bladders. It effectively prevented the reduction bladder blood flow (BBF) and the elevation in bladder weight, malondialdehyde levels (a marker of oxidative stress), proinflammatory cytokines and myeloperoxidase activity [77]. It has also been found that Eviprostat improved overactive bladder contractile response and down-regulated the increased levels of cytokines associated with cyclophosphamide-induced cystitis [110]. Clinical studies on Eviprostat demonstrated clea improvements in various parameters including IPSS, QoL score, and maximum and average urinary flow rates, increased prostatic volume, and reduced prostatic inflammation, without occurrence of any severe adverse effects [64, 104, 135].

##### Granu Fink femina

Granu Fink femina is obtained from seed oil from the combination of Uromedic pumpkin (cultivar of *Cucurbita pepo*), Rhus aromatica (fragrant sumach) bark extract, and *Humulus lupulus* (hop) cone extract. A clinical study [49] on 117 women (age: 21–78 year) with OAB showed that Granu Fink femina exhibited the ability to decrease urination frequency and reduce the average frequency of leakages and pad usage among patients. Notably, it significantly improved all aspects of quality of life related to OAB after just 1 week of use, with further enhancements observed at 6 and 12 weeks. In addition to these positive effects, the observed excellent tolerability profile makes it a very promising therapeutic option for OAB.

##### Kubiker

Kubiker (Naturmed, Montegranaro, FM, Italy) is a new complementary and alternative medicine (CAM), which has been proposed to treat OAB, containing vitamins (C and D), herbas (cucurbita maxima, capsicum annum, polygonum capsicatum) and amino acid L-Glutammina. In a randomized, controlled clinical study on 90 consecutive women (mean age 65 year; range 40–75) with symptoms of OAB, Kubiker was found to reduce daily micturitions, nocturia and episodes of urge incontinence, improve Patient Perception of Intensity of Urgency Scale (PPIUS), Overactive Bladder questionnaire Short Form (OAB-q SF) and Patient Global Impression of Improvement questionnaire (PGI-I), in a more effective level compared to Solifenacin Succinate [148].

##### Urox

Urox is a herbal mixure of 3 phytomedicines that have well established traditional uses, including *Crataeva nurvala* (used in Ayurvedic Medicine*)*, *Equisetum arvense* (an herbal remedy traced back to ancient Roman and Greek eras) and *Lindera aggregata* (wū yào 乌药, used in TCM). Efficacy of Urox on a range of bladder symptoms were studied clinically in a randomized, double-blind, placebo controlled trial with 150 participants. The treatment group exhibited a decrease in episodes of nocturia, symptoms of urgency, and total incontinence. After the treatment, notable enhancements in quality of life were reported, accompanied by minimal adverse effects [128].

## Discussion and conclusion

In recent years, there have been notable accomplishments in utilizing medicinal plants to treat OAB. This article reviews the current application as well as pharmological and clinical studies of medicinal plants or hebal formulations in OAB treatment, hoping to bring novel perspectives for the management and investigation of OAB.

Effective OAB therapy options are still very limited while patient compliance of therapeutic regimens is quite low. Alternative therapies are pursued by patients to mitigate symptoms of OAB. Accumulating evidence indicates the promising potential of medicinal plants and natural products in prevention and treatment of OAB, showing greater effectiveness and tolerability. Due to the differences in context and focus of natural product research and the complexity of ancient medicinal theories, the acceptability and clinical application of medicinal plants and natural products in Western medicine are greatly hindered. It is thus essential to conduct pharmacological evaluation using modern scientific strategies. Isolation and identification of the active ingredient(s) present in plants responsible for their biological or therapeutic activities are foundamentally important for understanding the underlying mechanisms. The present review shows that most of the plant extracts used to treat OAB are aqueous extracts, while the remaining are lipidic extracts or essential oils. Until now, only a few phytochemicals have been isolated and characterized for their pharmacological activities against OAB, and it is necessary to put more efforts into making progress in this aspect.

Limitation in study design is another obstacle for OAB herbal treatment. Firstly, the plant species utilized in the same treatment regimen may differ across publications, and it is important to note that a TCM herb may not consistently originate from a single plant species.Thus, the botanical Latin names of the plant species should be carefully confirmed. In addition, the quality of herbs/plants could directly influence their pharmacological activities and clinical effectiveness. Hence, it is crucial to accurately identify authentic herbs/plants. Geo-authentic plants are cultivated in their native regions, where the climate and ecological conditions are optimal for producing high-potency herbal products. The desired parts of these plants are harvested at the peak of their season and in a specific manner to ensure the preservation of their medicinal properties without compromise. To ensure the quality and safety of herbs, it is important to choose GAP suppliers with legitimate sources of geo-authentic herbs and GMP pharmaceutical manufacturers with established standardization procedures.

What’s more, various types of animal models for OAB are utilized, each with distinct applications and scopes [93]. Hence, how to choose an appropriate and suitable model for medicinal plants targeting different mechanisms needs further exploration.

Based on the review of existing evidence of medicinal plants, natural products and herbal formulations used in treating OAB, we summarized their possible interverntion mechanisms in the unrinary bladder (Fig. 2). Considering the complicated and elusive pathophysiology of OAB, the mechanisms of treatment of OAB with medicinal plants or natural products are complex and the exact mechanism of different agents remains to be fully elucidated and verified.

Previous studies indicated phytochemicals from natural sources has a higher pharmacokinetic stability than synthetic drugs in the body. Due to their nature as small molecular compounds, natural products exhibit rapid absorption and high permeability. It is suggested different plant extracts components have synergistic effects beneficial for their bioavailability. The precise mechanism of action and pharmacokinetic properties including absorption, distribution, metabolism and excretion (ADME) of biologically active ingredients should be thoroughly investigated. Moreover, the combination of traditional medicine or natural products with conventional chemical drugs has emerged as a novel avenue of research and their synergistic actions have been increasingly studied in recent years. Herbal formulations have a long-standing history of utilization in the management of OAB, both in traditional practices and contemporary times. In recent years, a plethora of scientific studies have been conducted to enhance our comprehension of the efficacy and underlying mechanisms of these formulations. The efficacy-enhancing and toxicity-reducing effects of different kinds of herbal combinations are an attractive strategy, considering the multifactorial pathogenesis of OAB and the limitations of current available therapeutic options.

In conclusion, this review yielded an evidence basis of various medicinal plants and natural products used for OAB treatment. However, none of these herbal drugs have advanced to become novel clinical therapy options for OAB. Consequently, there is still a significant demand for effective and well-tolerated phytopharmaceuticals in this regard. Promising new alternative herbal treatment strategies are emerging with high safety level and efficacy worthy of clinical application. There is a growing aspiration to foster the development of OAB management. Nevertheless, the existing cases of medicinal plants or natural products treating OAB are still relatively limited, and some of them have been only used in traditional ways without modern scientific research evidence. This review would serve as a reference basis for researchers investigating OAB and encourage more relevant pharmacological and clinical research on those understudied natural medicines. Clinical trials using phytochemicals or hebal formulations against OAB are still in infancy. Future rigorously designed controlled studies will allow the validation of their place in the therapeutic arsenal for OAB.

## Data Availability

All data are fully available without restriction.

## Abbreviations

AA: Acetic acid
Alpha, beta-MeATP: Alpha, beta-methylene ATP
AWR: Abdominal withdrawal reflex
BBF: Bladder blood flow
BE: Bethanechol
CYP: Cyclophosphamide
DHP: Dihydropyridine
DO: Detrusor overactivity
DSM: Detrusor smooth muscle
EFS: Electrical field stimulation
EMG: Electromyogram
ER: Endoplasmic reticulum
FDC: First detrusor contraction
ICAM-1: Intercellular adhesion molecule 1
IL-1β: Interleukin-1β
IPSS: International Prostate Symptom Scores
LUTS: Lower urinary tract symptoms
MCC: Maximal cytometric capacity
MS: Multiple sclerosis
NF-kB: Nuclear factor kappa B
NIH-CPSI: National Institute of Health—Chronic Prostatitis Symptom Index
NGF: Nerve growth factor
NOD: Neurogenic detrusor overactivity
OAB: Overactive bladder
OAB-q SF: Overactive Bladder questionnaire Short Form
OABSS: Overactive bladder symptom score
PGIC: Patient’s Global Impression of Change
PGI-I: Patient Global Impression of Improvement questionnaire
PPIUS: Patient Perception of Intensity of Urgency Scale
QOL: Quality of life
RA: Retinyl acetate
RTX: Resiniferatoxin
SCF: Stem cell factor
SLC17A9: Solute Carrier Family 17 Member 9
SNARE: Soluble N-ethylmaleimide-sensitive-factor attachment protein receptor
TCM: Traditional Chinese Medicine
TNF-α: Tumor necrosis factor- α
TRP: Transient receptor potential
TRPV1: Transient receptor potential V1
UPK3A: Uroplakin 3A
